# A standardized patient-centered characterization of the phenotypic spectrum of *PCDH19* girls clustering epilepsy

**DOI:** 10.1038/s41398-020-0803-0

**Published:** 2020-05-04

**Authors:** Kristy L. Kolc, Lynette G. Sadleir, Christel Depienne, Carla Marini, Ingrid E. Scheffer, Rikke S. Møller, Marina Trivisano, Nicola Specchio, Duyen Pham, Raman Kumar, Rachel Roberts, Jozef Gecz

**Affiliations:** 1 Adelaide Medical School, The University of Adelaide, Adelaide, SA Australia; 2 Department of Paediatrics and Child Health, University of Otago, Wellington, New Zealand; 3 Institute of Human Genetics, University Hospital Essen, University Duisburg-Essen, Essen, Germany; 4 Inserm U 1127, CNRS UMR 7225, Sorbonne Universités, UPMC Univ Paris, 06 UMR S 1127, Institut du Cerveau et de la Moelle épinière (ICM), F-75013 Paris, France; 5 Child Neurology and Psychiatric Unit, Pediatric Hospital G. Salesi, Ospedali Riuniti Ancona, Ancona, Italy; 6 Department of Medicine, Epilepsy Research Centre, The University of Melbourne, Austin Health, Melbourne, VIC Australia; 7 Department of Paediatrics, Royal Children’s Hospital, The University of Melbourne, Melbourne, VIC Australia; 8 The Florey Neuroscience and Murdoch Childrens Research Institutes, Melbourne, VIC Australia; 9 Department of Epilepsy Genetics and Personalized Medicine, The Danish Epilepsy Centre, Dianalund, Denmark; 10 Institute for Regional Health Services, University of Southern Denmark, Odense, Denmark; 11 Rare and Complex Epilepsy Unit, Department of Neuroscience, Bambino Gesù Children’s Hospital, IRCCS, Rome, Italy; 12 Robinson Research Institute, The University of Adelaide, Adelaide, SA Australia; 13 School of Psychology, The University of Adelaide, Adelaide, SA Australia; 14 Women and Kids, South Australian Health and Medical Research Institute, Adelaide, SA Australia

**Keywords:** Psychiatric disorders, Human behaviour

## Abstract

Protocadherin-19 (*PCDH19*) pathogenic variants cause an early-onset seizure disorder called girls clustering epilepsy (GCE). GCE is an X-chromosome disorder that affects heterozygous females and mosaic males, however hemizygous (“transmitting”) males are spared. We aimed to define the neuropsychiatric profile associated with *PCDH19* pathogenic variants and determine if a clinical profile exists for transmitting males. We also examined genotype- and phenotype–phenotype associations. We developed an online *PCDH19* survey comprising the following standardized assessments: The Behavior Rating Inventory of Executive Function; the Social Responsiveness Scale, 2nd edition; the Strengths and Difficulties Questionnaire; and the Dimensional Obsessive-Compulsive Scale. Genetic, seizure, and developmental information were also collected. The survey was completed by patients or by caregivers on behalf of patients. Of the 112 individuals represented (15 males), there were 70 unique variants. Thirty-five variants were novel and included a newly identified recurrent variant Ile781Asnfs*3. There were no significant differences in phenotypic outcomes between published and unpublished cases. Seizures occurred in clusters in 94% of individuals, with seizures resolving in 28% at an average age of 17.5 years. Developmental delay prior to seizure onset occurred in 18% of our cohort. Executive dysfunction and autism spectrum disorder (ASD) occurred in approximately 60% of individuals. The ASD profile included features of attention-deficit hyperactivity disorder. In addition, 21% of individuals met criteria for obsessive-compulsive disorder that appeared to be distinct from ASD. There were no phenotypic differences between heterozygous females and mosaic males. We describe a mosaic male and two hemizygous males with atypical clinical profiles. Earlier seizure onset age and increased number of seizures within a cluster were associated with more severe ASD symptoms (*p* = 0.001), with seizure onset also predictive of executive dysfunction (*p* = 4.69 × 10^−4^) and prosocial behavior (*p* = 0.040). No clinical profile was observed for transmitting males. This is the first patient-derived standardized assessment of the neuropsychiatric profile of GCE. These phenotypic insights will inform diagnosis, management, and prognostic and genetic counseling.

## Introduction

Protocadherin-19 (*PCDH19*) pathogenic variants cause an infantile onset seizure disorder called girls clustering epilepsy (GCE). GCE is an X-chromosome disorder with a unique expression pattern where heterozygous females and males with postzygotic somatic variants (“mosaic males”) are affected, but hemizygous (“transmitting”) males are unaffected. The hallmark of GCE is clusters of focal seizures, often coinciding with fever^1^. While the seizure semiology associated with GCE has been characterized^1–4^, the neuropsychiatric profile has not been well established.

*PCDH19* pathogenic variants are frequently associated with intellectual disability (ID) and psychiatric disturbances^3,5–7^. These neuropsychiatric comorbidities are highly heterogeneous, with ID ranging from mild to profound, and combinations of autistic, attention-deficit/hyperactive, obsessive, or aggressive features. The natural history of GCE shows that seizures become less frequent with age and cognition plateaus over time^6,8^. Psychiatric symptoms often increase with age and become the most disabling feature in some patients^3,8^. The specific cognitive deficits, and the severity and prevalence of comorbidities remain unknown.

In our systematic review and meta-analysis, we identified that executive dysfunction and hyperactive, autistic, and obsessive-compulsive features were most frequently reported in individuals with *PCDH19* pathogenic variants. We also showed that individuals with seizure onset before 12 months of age had more severe ID than those with seizure onset after 12 months^9^. A recent retrospective study validated this finding and also showed an association between earlier seizure onset and the presence of ASD^4^. Reviewed studies were typically based on small samples, lacked systematized approaches, and focused on only one clinical outcome.

Here we aim to define the neuropsychiatric profile associated with *PCDH19* pathogenic variants using standardized assessments that specifically target executive functions and symptoms associated with ASD, attention-deficit hyperactivity disorder (ADHD), and obsessive-compulsive disorder (OCD), and interrogate which factors predict the severity of these neuropsychiatric comorbidities.

## Method

### Study design and participants

The *PCDH19* survey was developed in English, Italian, and French using Survey Monkey (www.surveymonkey.com/) and was available from April 2017 through March 2019. Invited participants (*n* = 186) were parents or caregivers responding on behalf of individuals aged 2 years and over with a *PCHD19* variant or individuals over 10 years of age with a *PCDH19* variant who were able to self-report. Exclusion was based on our determination that the *PCDH19* variant was likely benign based on frequency in the general population and in silico assessment.

### Outcomes

We collected demographic and clinical information using the Epilepsy Questionnaire (EQ), which we developed based on literature review and discussion with health professionals (Supplementary materials). We assessed ASD using the Social Responsiveness Scale, second edition (SRS-2)^10^. As a French translation of the SRS-2 was not available, we also utilized the Social Communication Questionnaire (SCQ)^11^. Behavioral difficulties were assessed via the extended version of the Strengths and Difficulties Questionnaire (SDQ)^12^. We used the four-band categorization cutoff scores to assess symptoms of depression and anxiety (emotional problems scale), aggression (conduct problems and prosocial scales), ADHD (hyperactivity-inattention scale), and social deficits (peer problems and prosocial scales)^13^. SDQ categories were re-classified from “close to average” to “average”, “slightly raised/lowered” to “mild”, “high/low” to “moderate”, and “very high/very low” to “severe” to align our analysis with other assessed constructs. Executive dysfunction was assessed using the Behavior Rating Inventory of Executive Function (BRIEF)^14^. BRIEF inconsistency, negativity, and infrequency scales were included to assist in detecting bias associated with rating scales. We assessed OCD using the Dimensional Obsessive-Compulsive Scale (DOCS)^15^. The internal consistency of all assessments was acceptable: SRS-2 (Cronbach’s alpha = 0.97), SCQ (*α* = 0.86), SDQ scales *(M* = 0.73; Supplementary Table 1), BRIEF forms (*M* = 0.98), and DOCS (*α* = 0.96).

Translation of the EQ, survey scripts, and study material were performed and checked by either a professional translator or by an individual familiar with GCE and fluent in the relevant languages. Published authorized translations of the SRS-2, SCQ, SDQ, BRIEF, and DOCS were utilized. License agreements were obtained to reproduce assessments in an online format. The project was approved by the University of Adelaide Human Research Ethics Committee (H-2016-184). Electronic informed consent was obtained from all participants.

### Statistical analyses

All genetic variants were mapped to the longest isoform of the *PCDH19* mRNA (NM_001184880.1) and protein (NP_001171809.1) reference sequences (https://www.ncbi.nlm.nih.gov/). Variant annotation was based on nomenclature for the description of sequence variants (http://www.hgvs.org/mutnomen/). We identified whether the *PCDH19* variant had been previously reported, then assessed the pathogenicity of all novel variants based on gnomAD (http://gnomad.broadinstitute.org/) frequency and in silico prediction tools through the web-based ANNOVAR (http://wannovar.wglab.org/). Data were analyzed using Statistical Package for the Social Sciences version 25. ID severity was coded as previously described^9^. To test associations, we used a linear regression model and set statistical significance at *p* = 0.05. We used a deductive category analytical approach to evaluate qualitative data^16^.

## Results

Of the 122 completed surveys, 7 participants were excluded prior to, and 3 following, in silco PCDH19 variant assessment (Supplementary Table 2). Seven individuals reported secondary variants in other genes, which were not predicted to be likely pathogenic (Supplementary Table 3), so these participants remained in the analysis. Following exclusions, 112 individuals remained; including 97 heterozygous females (90 affected, 7 unaffected) and 15 males (6 hemizygous, 9 mosaic). The mean age at time of study (*n* = 112) was 17.6 years (SD = 15.6, range = 1.5–70 years).

The characteristic phenotype for *PCDH19* heterozygous females and mosaic males is epilepsy. Individuals without epilepsy were classified as “non-penetrant”. Of the 106 heterozygous females and mosaic males, there were eight non-penetrant individuals (seven females). Therefore, the penetrance of GCE in our cohort was 92%. The non-penetrant mosaic male (#63) was ascertained as they were the father of an affected female (#28). Of the seven non-penetrant females (#49, #56, #58, #62, #93, #97, #99), two (#58, #93) had a single febrile seizure^17^. Interestingly, our cohort included two symptomatic hemizygous males, one with epilepsy (#39) and one with ASD and no epilepsy (#47). The remaining four hemizygous males were classified as “transmitting”, as they were the asymptomatic fathers of affected females.

### PCDH19 DNA variants

**Fig. 1:**
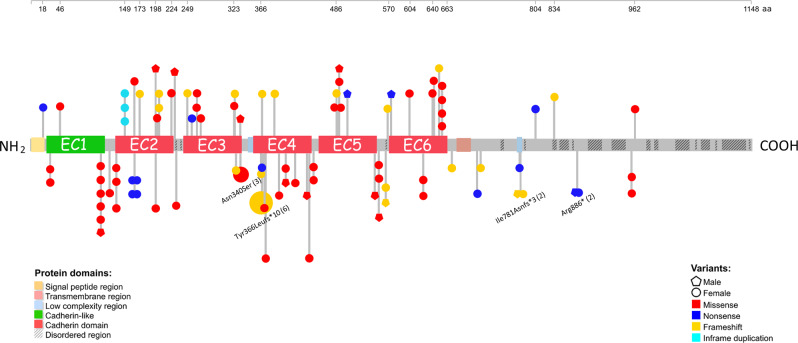
Lollipop plot illustrating all PCDH19 variants in our cohort (*n* = 102) excluding whole gene deletions and splicing variants. Lollipop size is exponentially proportional to the number of times the variant has been observed in unrelated individuals (recurrent). At a given locus, the number of lollipops represents the number of related individuals with that variant, with the exception of Asn340Ser (2 unrelated families and one sporadic case) where this is illustrated in text. Unpublished (novel) variants (*n* = 34) are located above the protein and published variants (*n* = 32) are below the protein.

There were 70 unique *PCDH19* variants, of which 35 were novel. Variants included 25 frameshift, 14 nonsense, 60 missense, 3 in-frame duplications (Fig. 1), 6 whole gene deletions and 4 splicing. Almost half (54/112) arose de novo, with 18 paternal, 21 maternal, or 19 of unknown origin. There were four recurrent variants: Arg886* (2), Ile781Asnfs*3 (2), Asn340Ser (3), and Tyr366Leufs*10 (6). The Ile781Asnfs*3 variant is newly identified as recurrent (Supplementary Materials).

### Development

Early development was ascertained only for surveys completed by a parent or caregiver (*n* = 83; Supplementary Table 4a). Developmental delay occurred in 50/83 (60%). The average age of first developmental concern was 12.8 months (SD = 11.1, range = 1–48 months). All developmental domains were affected, however, communication (24/50) and motor skills (22/50) were most affected. Delay prior to the onset of seizures occurred in 15/49 individuals (31%). For one individual (#47), developmental delay was noted at 12 months in the absence of seizures. Regression occurred in 37/83 (46%), and regression following a seizure cluster occurred in 30/37 (81%).

The intellect of the entire cohort was based on pre-existing diagnosis. Information regarding the degree of ID was not available for one individual (#22), therefore they were excluded from this analysis. Normal intellect was reported in 62/111 (56%), borderline in 4/111 (3.5%), mild ID in 20/111 (18%), moderate ID in 9/111 (8%), severe ID in 14/111 (12.5%), and profound ID in 2/111 (2%) of our patient cohort (Supplementary Table 4b).

### Seizures

**Fig. 2:**
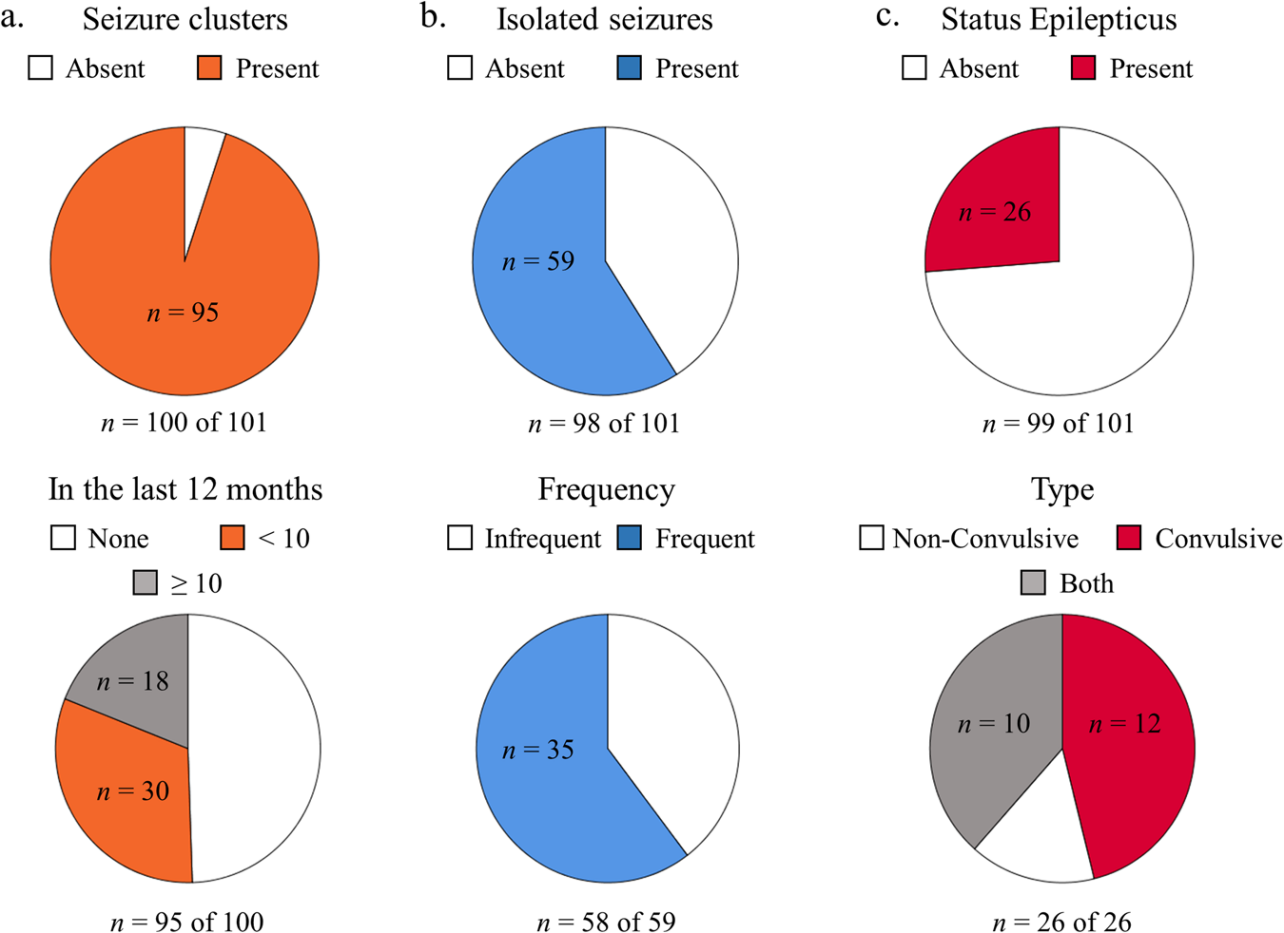
Seizure characteristics. Seizure characteristics include: (**a**) proportion of individuals with seizure clusters and number of clusters in the last 12 months; (**b**) proportion of individuals with isolated seizures and the frequency of isolated seizures (infrequent refers to less than yearly and frequent to occurrence ranging from daily to yearly); and (**c**) proportion of individuals with episodes of status epilepticus and type of status epilepticus.

Age at seizure onset for heterozygous females (*n* = 90) ranged from 1.5 to 60 months (*M* = 12.2, SD = 9.27, median = 10 months) and for mosaic males (*n* = 8) from 5 to 96 months (*M* = 20.6, SD = 30.9, median = 9 months). The most common age at onset was 8 months (14%, *n* = 98). Cluster duration (*n* = 93) ranged from 1 to 24 days (*M* = 4.61, SD = 4.29) and the average number of seizures within a cluster ranged from 2 to 100 (*M* = 15.53, SD = 13.99), with most clusters lasting for 2 days and averaging 10 seizures per cluster (Fig. 2; see also Supplementary Materials).

Seizure offset for individuals aged 11 years and over (based on the youngest reported seizure offset age) had occurred in 28% of our cohort. Age at seizure offset ranged from 11 to 38 years (*M* = 17.6, SD = 7.43). Epilepsy is classified as resolved when an individual has been seizure free for 10 years, with the last five years free from antiepileptic medication^17^. Sixteen individuals (16%), aged 22 to 52 years (*M* = 32.8, SD = 8.55) were seizure-free for at least 10 years. Eleven of the 16 were taking antiepileptic medication. For the five individuals who were seizure-free without medication, age at seizure offset ranged from 11 to 38 years (*M* = 20.7, SD = 10.8).

### Neuropsychiatric comorbidities

#### Autism spectrum disorder

**Fig. 3:**
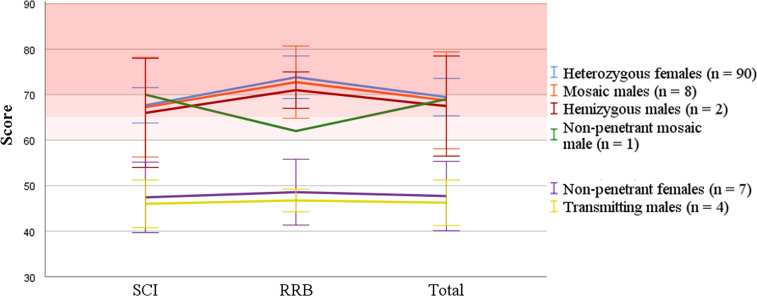
Average (±2 SEM) SRS-2 total and DSM-5 domain t scores by group. Darkening shades of red correspond to increasing degrees of severity. SCI, social communication and inhibition; RRB, restricted interests and repetitive behavior.

All participants completed either the SRS-2 (*n* = 104) or the SCQ (*n* = 8). Total average SRS-2 scores fell in the normal range for transmitting males and non-penetrant females and in the clinical range for all other groups (Fig. 3). Total SRS-2 scores were in the clinical range in 68% of females (56/82), including one non-penetrant female (#58), 75% of mosaic males (6/8) including the non-penetrant male (#63), and both hemizygous males (#39, #47). There was no sex difference in severity of ASD (*p* = 0.781; Supplementary Fig. 1). The SCQ average total score ranged from 8 to 32 (*M* = 18.5, SD = 8.1), and fell in the clinical range for five individuals (63%).

#### Behavioral assessment

The SDQ was completed by parents/caregivers of individuals aged between 2 and 17 years (*n* = 73). Elevated scores were observed across all SDQ domains, with the hyperactivity-inattention, peer problems, and prosocial behavior domains most severely affected (Supplementary Table 5a, b). Impact scores were in the very high range for approximately 75% of participants (Supplementary Table 5c). This was further supported by 65% of respondents endorsing behavior as the most challenging aspect each day.

#### Executive dysfunction

One participant (#87) was excluded due to missing data (*n* = 111). For individuals aged between 2–4 years (*n* = 17), BRIEF total global executive composite *t* scores (GEC*t*) fell within the clinical range for heterozygous females (*M* = 68.1) and mosaic males (*M* = 62.5). Inhibit (*M* = 68.5) and working memory (*M* = 70.9) domains were the most affected for females, with working memory also elevated for males (*M* = 72.5). For individuals aged between 5-17 years (*n* = 57), GEC*t* scores fell within the clinical range for heterozygous females (*M* = 73.2) and mosaic males (*M* = 67.0), and within the normal range for one hemizygous male (#39). Elevation in all domains were observed for females, with the exception of organization of materials. Shift, working memory and plan/organize were elevated for males. For individuals aged 18 and over (*n* = 37), GEC*t* scores fell in the normal range for all but one hemizygous male (#47).

**Fig. 4:**
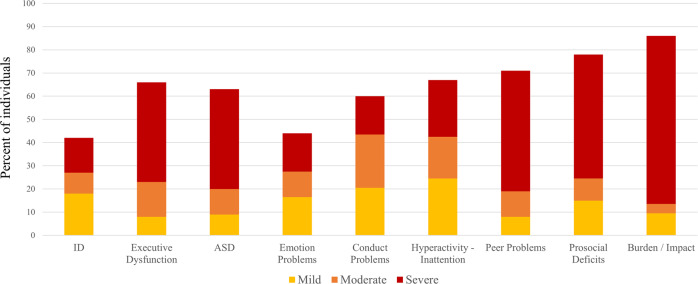
The percentage of each comorbidity associated with PCDH19 variants. ID intellectual disability, ASD autism spectrum disorder (severity based on SRS-2 t scores only). Severe ID included two individuals with profound ID. DOCS scores omitted as no cutoff exists for severity.

Average scores in the shift domain were elevated for most groups, including one hemizygous male (#47) and the non-penetrant mosaic male (#63; Supplementary Fig. 2). Overall, executive dysfunction was observed in 72% of heterozygous females (64/89), 50% of mosaic males (4/8), one non-penetrant female (1/7) and one hemizygous male (1/2). Again, no difference was observed between males and females (*p* = 0.482; Supplementary Fig. 1). Inconsistency, negativity, and infrequency scores were in the acceptable range. Figure 4 summarizes the neuropsychiatric profile of our cohort.

#### Obsessive compulsive disorder

The DOCS was administered only to individuals who could complete the assessment themselves (*n* = 29). One participant (#56) was excluded due to missing responses (*n* = 28). Six individuals (21%), attained a total score that was consistent with a possible OCD diagnosis. All six were heterozygous females, including one non-penetrant female (#58). All transmitting males scored in the normal range. See Supplementary Table 6 for a summary of all neuropsychiatric measure descriptive statistics by group.

### Predicting the severity of neuropsychiatric comorbidities

**Fig. 5:**
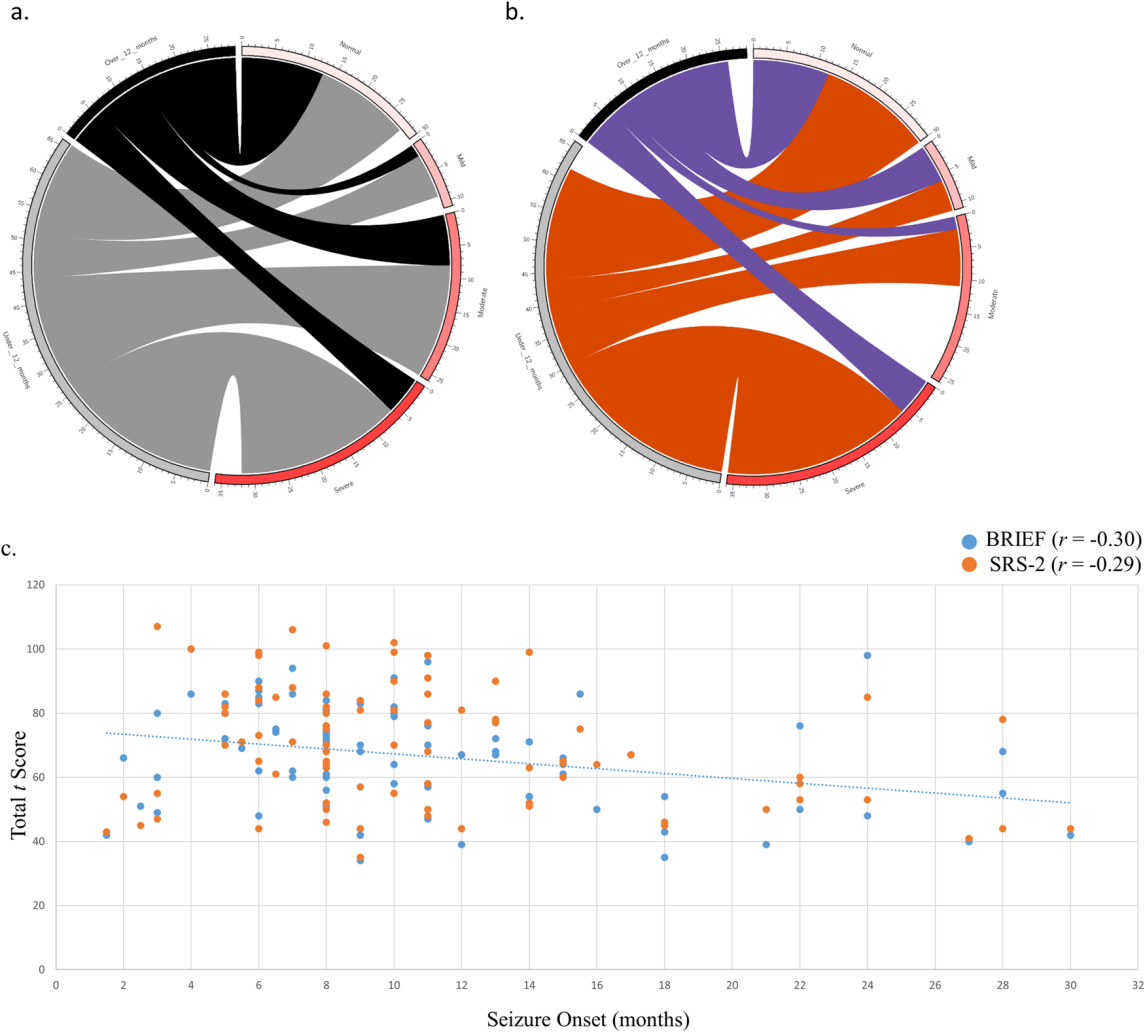
Circos and scatterplot illustrating phenotype–phenotype association. **a** The variable cognitive profile of GCE (*n* = 95) against age at seizure onset: ≤12 months, represented by grey links (*n* = 66) and >12 months, represented by black links (*n* = 29). **b** The variable ASD profile of GCE (*n* = 88) against age at seizure onset: ≤12 months, represented by orange links (*n* = 62) and >12 months, represented by purple links (*n* = 26). Axes show the number of individuals in each category. **c** A moderate negative association between age at seizure onset and clinical outcome, as measured by the BRIEF (*n* = 93) and SRS-2 (*n* = 86).

Five outliers (#39, #50, #58, #64, & #112) were removed prior to statistical analyses due to seizure onsets 2 standard deviations above the mean. There was a significant negative association between age at seizure onset and ID for novel variants (*p* = 0.007) as well as for the entire cohort (*p* = 0.010). For every one month increase in seizure onset age, there was a 0.07 decrease in average ID severity, controlling for age at time of study (estimate = −0.07, 95% CI: −0.12, −0.02). There was a significant negative association between age at seizure onset and executive dysfunction for novel variants (*p* = 0.044) as well as the entire cohort (*p* = 4.69 × 10^−4^; Fig. 5a). On average, increasing seizure onset age by 1 month was associated with a 0.82 decrease in average GEC*t* scores, controlling for age at time of study (estimate = −0.82, 95% CI: −1.27, -0.37). For the ASD analysis, SRS-2 and SCQ total scores were converted to *z* scores and combined. Seizure onset age was significantly associated with ASD (*p* = 0.001; Fig. 5b) and prosocial behavior (*p* = 0.040). On average, increasing seizure onset age by 1 month was associated with a 0.50 decrease in SCQ/SRS-2 combined scores (estimate = −0.50, 95% CI: −0.08, -0.02) and a 0.13 increase in prosocial behavior scores (estimate = 0.13, 95% CI: 0.01, 0.25), controlling for age at time of study. No significant association was observed for the hyperactivity-inattention and peer problems scales.

We hypothesized that seizure activity (operationalized as the average number of seizures per day in a cluster) combined with onset would strengthen these associations. An additional outlier (#26) was removed from this analysis. For a one-way analysis of variance, seizure onset was categorized as “early” (≤12 months) or “late” (>12 months)^9^ and seizure activity was categorized as “mild” (≤15 seizures/day in a cluster) or “severe” (>15 seizures/day in a cluster) based on the group average. Seizure activity was associated with executive dysfunction, *F*_3,81_ = 4.71, *p* = 0.004, ASD, *F*_3,81_ = 7.45, *p* = 1.82 ×10^−4^, and prosocial behavior, *F*_3,66_ = 3.10, *p* = 0.033. We predicted that the greatest phenotypic difference would be observed between individuals with late onset/mild seizure activity and individuals with early onset/severe seizure activity. Our prediction was supported for all outcomes: executive dysfunction (Supplementary Fig. 3a; *t*_81_ = −3.45, *p* = 0.001), ASD (Supplementary Fig. 3b; *t*_81_ = −4.66, *p* = 1.2 × 10^−5^), and prosocial behavior (*t*_66_ = −2.92, *p* = 0.005), with an earlier age at seizure onset combined with severe seizure activity being associated with more severe outcomes. An examination of the means revealed that seizure onset was more strongly associated with cognitive outcomes whereas seizure activity was more strongly associated with psychiatric outcomes (Supplementary Table 7).

Given that 50% of our cohort had novel variants, we wanted to exclude that the clinical outcome for published cases would be more severe than that of unpublished cases due to selection or admission bias^18^. We investigated this via *t* tests and found no statistically significant difference for either executive dysfunction (*M*_published_ = 67.5; *M*_unpublished_ = 62.2) or standardized ASD symptom severity (*M*_published_ = 0.14; *M*_unpublished_ = −0.24). The SDQ and DOCS were excluded, as fewer individuals had scores on these measures. Consistent with our previous finding, there were no notable genotype-phenotype associations (Supplementary Table 8).

## Discussion

We performed the first comprehensive patient-derived standardized assessment of *PCDH19*-variant individuals, including males (both germline and mosaic) and non-penetrant females. Females with GCE are typically described as having normal early development and regressing in infancy^8,19,20^. We found that delayed development prior to seizure onset occurred in 18% of individuals, replicating observations in two smaller studies^2,3^. This may provide scope for early detection, especially for siblings of affected individuals. Seizures occurred in clusters in 94% of individuals. We believe the hallmark features of GCE to be threefold: (1) focal seizure clusters with affective semiology^1^, often triggered by fever; (2) seizure onset at 8 months of age; and (3) predominantly affecting females. The molecular diagnosis for many infantile neurodevelopmental disorders including epilepsy occurs at a mean age of 3 years, which represents delays of months to years for patients with pathogenic variants^21^. Early clinical identification of GCE will result in earlier molecular diagnosis and may impact outcome by allowing optimization of both seizure management and developmental progress.

Overall, 69 out of 112 (62%) individuals met criteria for ASD. GCE has previously been associated with ASD^2,5^, suggesting that this genetic etiology underlies epilepsy, ASD, and ID. The frequency of ASD in our cohort was lower than previous estimates, but may be more accurate given the much smaller sample sizes and lack of systemized approaches in these studies^3–6^. We found that executive dysfunction occurs in 70 out of 111 (63%) individuals. Of these, 62 (89%) individuals also met criteria for ASD. It has been posited that the social and non-social deficits observed in ASD stem from deficits in executive functions and might explain the co-occurrence of these disorders^22^.

The SDQ revealed that peer problems and prosocial behavior were the most affected domains. This is expected given the high proportion of individuals meeting criteria for ASD. Most individuals who met criteria for ASD, also scored high on the BRIEF inhibit and shift subscales. The inhibit domain is relatively preserved in ASD, yet impaired in ADHD whereas shift domain deficits are characteristic of ASD rather than ADHD^23^. The ASD group with elevated inhibit (ADHD-like) and shift (ASD-like) scores also scored very high on hyperactivity-inattention (see Supplementary Output). This may represent an ASD profile with features of ADHD or a general deficit in executive functions that underlies these comorbidities. SDQ impact assesses chronicity, distress, social impairment, and burden for others. Most (75%) scores were in the very high range. This finding, combined with qualitative accounts, supports reports that psychiatric comorbidities become the most concerning feature in GCE.

Consistent with a recent report^3^, 21% of our cohort had obsessive-compulsive symptomatology revealed by the DOCS. This may be an accurate estimate or may reflect the similarities between OCD and other disorders, such as ASD. Only one of the six individuals meeting criteria for OCD also met criteria for ASD in conjunction with a moderately elevated shift score (#58), suggesting that OCD is distinct from ASD in GCE.

We demonstrated that the clinical profile for heterozygous females and mosaic males is the same. We describe seven non-penetrant females and one male. This non-penetrance may reflect absence of mosaicism in the brains of these individuals, consistent with the cellular interference model^24,25^. We confirmed there is no clinical profile for transmitting males, although identified two hemizygous males with ASD in addition to executive dysfunction (#42) or seizures (#39). If their phenotypes are due to their *PCDH19* variants, these findings will expand the phenotypic spectrum; however, they may be due to additional genetic or environmental factors.

Consistent with our previous meta-analysis, earlier seizure onset age was associated with more severe ID. We also showed that earlier seizure onset age predicted greater executive dysfunction, prosocial behavior, and ASD severity, and that increased seizure activity strengthened these associations. It could be that earlier and more frequent seizures cause more adverse outcomes^26^, or the *PCDH19* variant is enhanced by polygenic or epigenetic burden^27^.

Although the frequency of comorbid ASD in the GCE population is likely to be correct, we are limited by inherent difficulties in delineating ASD from other impairments. For example, parent reports are not entirely reliable in discriminating children with ASD from those with language impairment^28^. An assessment of language was beyond the scope of this study. As language delay may be associated with GCE^9^, future work should incorporate a formal assessment of ASD and language impairment.

Biases also exist with self-reported data^29^. Validity checks within the BRIEF addressed these biases to some extent. Retrospective accounts are inherently less reliable than direct observation of behavior or events. However, standardized assessments are particularly useful in contexts such as these, as they minimize any potential confounds that are likely to emerge as a result of subjectivity and different administrators or methodology^30^.

GCE is a distinctive epilepsy with early onset of seizure clusters, with or without ID. We show that individuals with *PCDH19* pathogenic variants may have associated executive dysfunction, ASD, ADHD, and OCD, thus characterizing the neuropsychiatric profile of GCE. Our data show that approximately 20% of individuals have developmental delay prior to seizure onset. We confirm the association between earlier seizure onset age and more severe ID and demonstrate that the association with earlier seizure onset age extends to neuropsychiatric comorbidities. We also demonstrate an association between increased seizure frequency and poorer clinical outcomes. We show that the clinical profile for heterozygous females and mosaic males is the same and describe seven non-penetrant females and one male. We also show that there is no clinical profile for transmitting males, but identify two affected hemizygous males. These phenotypic insights will lead to better diagnosis and management of GCE.

## Supplementary information

Suppl Material Epilepsy Questionnaire

Supplementary Figures

Supplementary Materials

Supplementary Seizure Data

Supplementary Tables

Supplementary Variant Data
