# Supplementary material for: A standardized patient-centered characterization of the phenotypic spectrum of PCDH19 girls clustering epilepsy
Source: Transl Psychiatry. 2020 May 4;10:127. doi: 10.1038/s41398-020-0803-0 (PMC7198503; doi:10.1038/s41398-020-0803-0)
Supplement: Supplementary file 1 — Suppl Material Epilepsy Questionnaire [file 41398_2020_803_MOESM1_ESM.pdf]

THE UNIVERSITY of ADELAIDE

EPILEPSY QUESTIONNAIRE (EQ)

DEMOGRAPHICS

YOUR DETAILS: Please enter your details, as outlined below.
YOUR CHILD'S DETAILS: Please enter your child's details, as outlined below.
THE INDIVIDUAL'S DETAILS: Please enter the details of the individual you are responding on behalf of, as outlined below.

***First name:**

***Surname:**

***Date of birth: dd/mm/yyyy**

***Sex:**
☐ Male
☐ Female

Race:
☐ African
☐ Asian
☐ Caucasian
☐ Indigenous
☐ Other (please specify)

Country of birth:

Mother's age (in years):

Father's age (in years):

Age of other parent (in years):

Relationship to individual:

Age (in years) of mother (if known/applicable):
Age (in years) of father (if known/applicable):

DEVELOPMENTAL DETAILS

The following questions relate to your development.
The following questions relate to your child's development.
The following questions relate to the development of the individual you are responding on behalf of.

*Have you had a neurocognitive assessment?
*Has your child had a neurocognitive assessment?
*Has the individual had a neurocognitive assessment?

☐ YES
☐ NO
☐ UNSURE

If you are happy to share your neurocognitive assessment, please attach it here.
If you are happy to share their neurocognitive assessment, please attach it here.
Choose File

PREVIOUS DEVELOPMENT

The following questions relate to your child's previous development.

Is your child developmentally normal?

☐ YES
☐ NO
☐ UNSURE

At what age (in months) did you first notice they were not developing as they should?

What aspects of their development were affected?

Did your child lose any developmental skills that they previously had?

☐ YES
☐ NO
☐ UNSURE

How many times did this happen?

☐ Once
☐ Twice
☐ Three times
☐ Four times
☐ Five times or more

Was this associated with a cluster of seizures?

☐ YES
☐ NO
☐ UNSURE
☐ N/A

Was this associated with seizures lasting longer than 30 minutes?

☐ YES
☐ NO
☐ UNSURE
☐ N/A

CURRENT DEVELOPMENT

The following questions relate to your development at the present time.
The following questions relate to your child's development at the present time.
The following questions relate to the individual's development at the present time.

What year/grade are you in at school?
What year/grade is your child in school?

☐ Preschool/Nursery school/Day care
☐ Kindergarten/Reception
☐ Year 1
☐ Year 2
☐ Year 3
☐ Year 4
☐ Year 5
☐ Year 6
☐ Year 7
☐ Year 8
☐ Year 9
☐ Year 10
☐ Year 11
☐ Year 12
☐ Not currently attending school
☐ Never attended school

What is the highest level of education you have attained?
What is the highest level of education they attained?

☐ Attended some school
☐ Graduated high/secondary school
☐ Diploma
☐ Undergraduate degree
☐ Postgraduate degree
☐ Have never attended school

Are they able to do age appropriate work?
Are they able to do age-appropriate tasks?

☐ YES
☐ NO
☐ UNSURE

Do they require assistance/help with completing age-appropriate work?
Do they require assistance/help with completing age-appropriate tasks?

☐ YES
☐ NO
☐ UNSURE

*Have you been diagnosed with an intellectual disability?
*Has your child been diagnosed with an intellectual disability?
*Have they been diagnosed with an intellectual disability?

☐ YES
☐ NO
☐ UNSURE

*What level of intellectual disability have you been diagnosed with?
*What level of intellectual disability has your child been diagnosed with?
*What level of intellectual disability have they been diagnosed with?

☐ Borderline
☐ Mild
☐ Moderate
☐ Severe
☐ Profound
☐ Unsure

Which of these most accurately describes your child's language proficiency?
Which of these most accurately describes the individual's language proficiency?

☐ Pre-verbal (e.g., coos and makes pleasure sounds)
☐ Distinct consonant/vowel sounds (e.g., babbles in a speech-like way)
☐ Single word utterances (e.g., "Hi", "dog", "Dada", or "Mama")
☐ Two word utterances (e.g., "Where kitty?" or "more cookie")
☐ Two or three word phrases (i.e., speaks in a way that is understood by family members and friends)
☐ Four or more word sentences (i.e., speaks easily without having to repeat syllables or words)
☐ Fluency in native language (i.e., communicates easily with other children and adults)

MUTATION DETAILS

The next questions relate to your diagnosed mutation.
The next questions relate to your child's diagnosed mutation.
The next questions relate to the individual's diagnosed mutation.

*What is your diagnosed mutation? *E.g., c.1019A>G or p.D340N*
*What is your child's diagnosed mutation? *E.g., c.1019A>G or p.D340N*
*What is their diagnosed mutation? *E.g., c.1019A>G or p.D340N*
If you are unsure, just type "unsure."

*Please select the option that reflects the inheritance of your mutation.

☐ Only I have the mutation
☐ I have the mutation but neither parent has been tested
☐ Both my mother and I have the mutation
☐ Both my father and I have the mutation
☐ Unsure

*Please select the option that reflects the inheritance of your child's mutation.

☐ Only my child has the mutation
☐ My child has the mutation but neither parent has been tested
☐ My child and I have the mutation
☐ My child and his/her other parent have the mutation
☐ Unsure

*Please select the option that reflects the inheritance of their mutation.

☐ Only the individual has the mutation
☐ The individual has the mutation but neither parent has been tested
☐ The individual and their mother have the mutation
☐ The individual and their father have the mutation
☐ Unsure

*Have you been diagnosed with any additional mutations?
*Has your child been diagnosed with any additional mutations?
*Has the individual been diagnosed with any additional mutations?

☐ YES
☐ NO
☐ UNSURE

Please provide details of any additional mutations that you have been diagnosed with. *E.g., SCN1A, c.2837A>G or p.R466H*
Please provide details of any additional mutations that your child has been diagnosed with. *E.g., SCN1A, c.2837A>G or p.R466H*
Please provide details of any additional mutations the individual has been diagnosed with. *E.g., SCN1A, c.2837A>G or p.R466H*

SCREENING

Has your child ever had a seizure?
Has the individual ever had a seizure?
Have you ever had a seizure?

☐ YES
☐ NO

MEDICATION DETAILS

The next questions relate to your seizure medication.
The next questions relate to your child's seizure medication.
The next questions relate to the individual's seizure medication.

Please select all the medication that you have **previously taken** for your seizures.
Please select all the medication that your child has **previously taken** for their seizures.
Please select all the medication that the individual has **previously taken** for their seizures.

Please select all the medication that you are **currently taking** for your seizures.
Please select all the medication that your child is **currently taking** for their seizures.
Please select all the medication that the individual is **currently taking** for their seizures.

☐ Acetazolamide (e.g., Diamox)
☐ Carbamazepine (e.g., Tegretol)
☐ Cannabidiol (e.g., Sativex)
☐ Clobazam (Onfi, Frisium)
☐ Clonazepam (e.g., Klonotin)
☐ Clorazepate (Tranxene)
☐ Ethosuximide (Zarontin)
☐ Gabapentin (e.g., Neurontin)
☐ Lacosamide (Vimpat)
☐ Lamotrigine (e.g., Lamictal)
☐ Levetiracetam (Keppra)
☐ Nitrazepam (e.g., Alodorm)
☐ Oxcarbazepine (Oxtellar, Trileptal)
☐ Perampanel (Fycompa)
☐ Phenytoin (e.g., Dilantin)
☐ Piracetam (e.g., Nootropil)
☐ Potassium Bromide (Dibro-Be mono)
☐ Primidone (Mysoline)
☐ Pyridoxine/Vitamin B6 (e.g., Neuro-K)
☐ Retigabine/Ezogabine (Potiga, Trobalt)
☐ Rufinamide (Banzel, Inovelon)
☐ Stiripentol (Diacomit)
☐ Sultiame/Sulthiame (Ospolot)
☐ Tiagabine (Gabitril)
☐ Topiramate (Topamax)
☐ Valproate/Valproic Acid (e.g., Depakote)
☐ Vigabatrin/Gamma Vinyl GABA (Sabril)
☐ Zonisamide (Zonegran)
☐ Other (please specify)

*Have your seizures been controlled with medication for a period of time?
*Have your child's seizures been controlled with medication for a period of time?
*Have the individual's seizures been controlled with medication for a period of time?
☐ YES
☐ NO

Are you currently seizure-free?
Is your child currently seizure-free?
Is the individual currently seizure-free?
☐ YES
☐ NO

Please provide details of the medication or medications that have been successful in controlling your seizures. Please provide the duration (in months) that you have remained seizure free in brackets following each medication listed. *E.g., Valproate (13 months), Lamotrigine (5 months)*

Please provide details of the medication or medications that have been successful in controlling your child's seizures. Please provide the duration (in months) that your child remained seizure free in brackets following each medication listed. *E.g., Valproate (13 months), Lamotrigine (5 months)*

Please provide details of the medication or medications that have been successful in controlling the individual's seizures. Please provide the duration (in months) that they remained seizure free in brackets following each medication listed. *E.g., Valproate (13 months), Lamotrigine (5 months)*

SEIZURE DETAILS

The next questions relate to your seizures.
The next questions relate to your child's seizures.
The next questions relate to the individual's seizures.

*At what age (in months) did you begin having seizures?
*At what age (in months) did your child begin having seizures?
*At what age (in months) did they begin having seizures?
If you are unsure, just type "unsure."

How many hospital admissions for seizures have you had?
How many hospital admissions for seizures has your child had?
How many hospital admissions for seizures have they had?
☐ 0
☐ 1
☐ 2
☐ 3
☐ 4
☐ 5+
☐ Unsure

Have you been admitted to ICU as a result of seizures?
Has your child been admitted to ICU as a result of seizures?
Have they been admitted to ICU as a result of seizures?
☐ YES
☐ NO

*Have you ever had a seizure lasting longer than 30 minutes?
*Has your child ever had a seizure lasting longer than 30 minutes?
*Have they ever had a seizure lasting longer than 30 minutes?

☐ YES
☐ NO
☐ UNSURE

How often do you have seizures that last longer than 30 minutes?
How often does your child have seizures that last longer than 30 minutes?
How often do they have seizures that last longer than 30 minutes?

☐ Daily
☐ Weekly
☐ Monthly
☐ Yearly
☐ Other (please specify)

How long do these seizures last? *Multiple options may be selected.*

☐ 30-40 minutes
☐ 40-50 minutes
☐ 50-60 minutes
☐ 60+ minutes

Are these long seizures convulsive (limbs shaking)?

☐ YES
☐ NO
☐ UNSURE

Are these long seizures non-convulsive?

☐ YES
☐ NO
☐ UNSURE

*Do your seizures cluster (many seizures occur together then there are long periods of time with no seizures)?
*Do your child's seizures cluster (many seizures occur together then there are long periods of time with no seizures)?
*Do their seizures cluster (many seizures occur together then there are long periods of time with no seizures)?

☐ YES
☐ NO
☐ UNSURE

What is the average number of days a cluster lasts?

What is the average number of seizures per day in a cluster?

How many seizure clusters have you had in the last 12 months?
How many seizure clusters has your child had in the last 12 months?
How many seizure clusters have they had in the last 12 months?

* Do you ever experience isolated seizures (not in a cluster)?
*Does your child ever experience isolated seizures (not in a cluster)?
*Do they ever experience isolated seizures (not in a cluster)?
☐ YES
☐ NO
☐ UNSURE

When you have experienced isolated seizures, how often did they occur?
When they have experienced isolated seizures, how often did they occur?
☐ Daily
☐ Weekly
☐ Monthly
☐ Yearly
☐ Other (please specify)

When was your last seizure?
When was your child's last seizure?
When was the individual's last seizure?

FEEDBACK

What do you find to be most challenging each day?
As a parent, what do you find to be most challenging each day?
As someone who is close to this individual, what do you find to be most challenging each day?

What do you find to be the most helpful in responding to these challenges?

Is there any other information that you wish to share (such as, seizures are commonly associated with temperature instability)?

*Response required for question branching
