# Supplementary material for: A standardized patient-centered characterization of the phenotypic spectrum of PCDH19 girls clustering epilepsy
Source: Transl Psychiatry. 2020 May 4;10:127. doi: 10.1038/s41398-020-0803-0 (PMC7198503; doi:10.1038/s41398-020-0803-0)
Supplement: Supplementary file 2 — Supplementary Figures [file 41398_2020_803_MOESM2_ESM.pdf]

Supplementary Figures

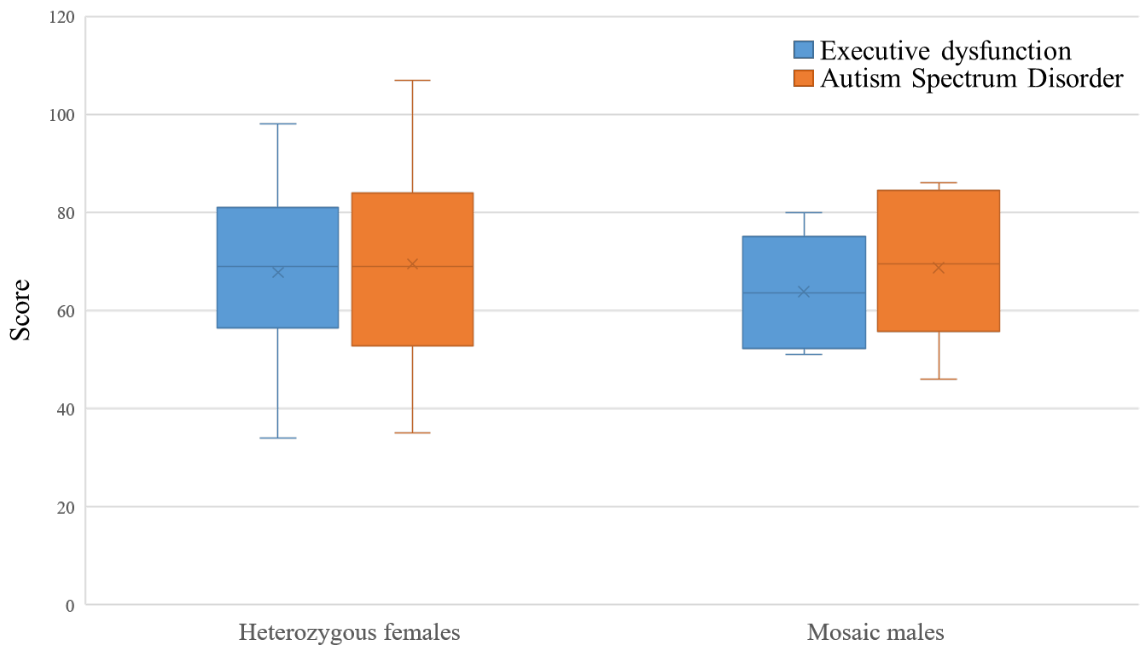

Figure 1. Boxplots illustrating average total BRIEF *t* scores (blue) for females (*n* = 89) and males (*n* = 8) and average total SRS-2 *t* scores (orange) for females (*n* = 82) and males (*n* = 8).

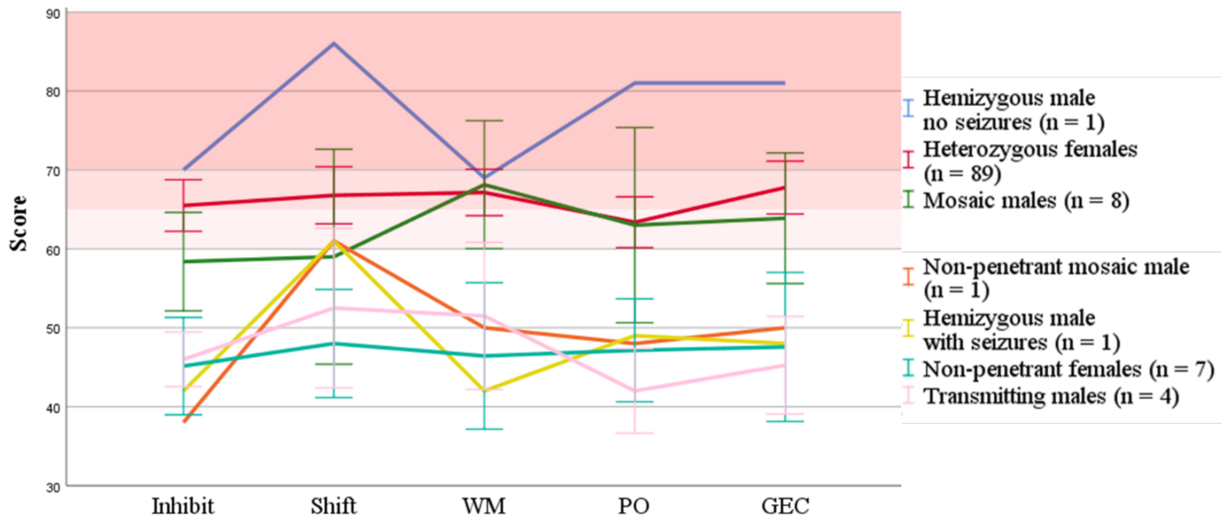

Figure 2. Average (±2 SEM) BRIEF total (GEC) and subscale *t* scores. Darkening shades of red correspond to increasing degrees of severity. *WM* working memory, *PO* plan/organize, *GEC* global executive composite.

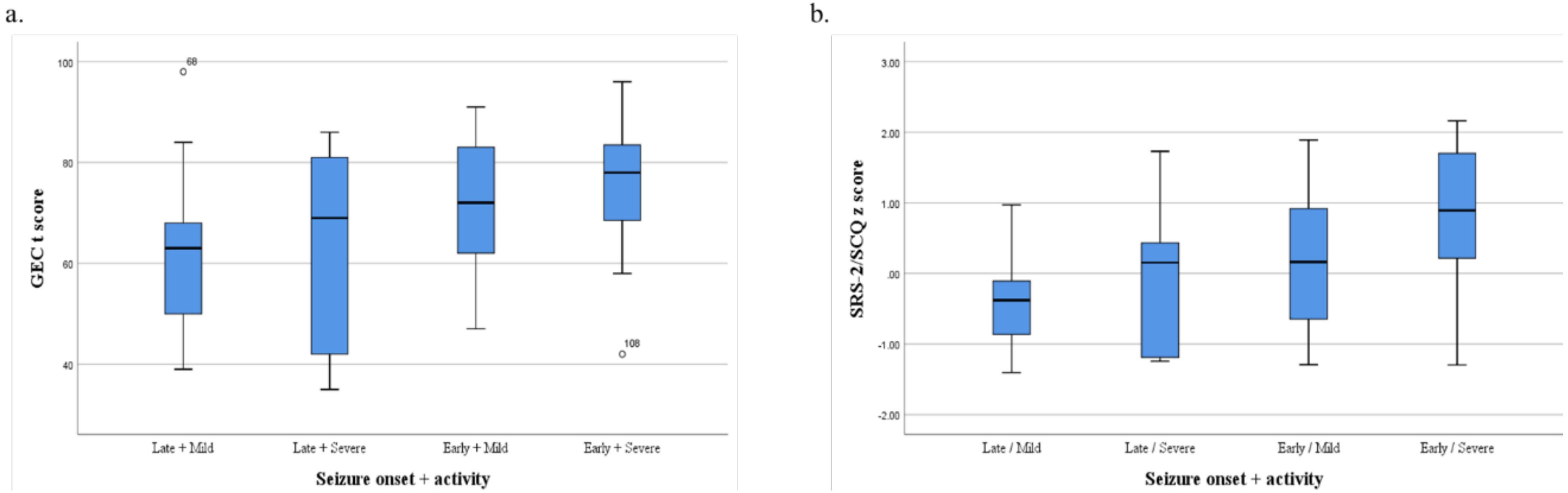

Figure 3. Association between seizure onset, seizure activity and clinical outcome: a) executive dysfunction measured by the BRIEF and b) ASD outcome measured by the SRS-2 or SCQ (scores converted to z scores for the analysis). *Early* = ≤12 months age at onset, *Late* = >12 months seizure onset, *Mild* = ≤15 average seizures/day, *Severe* = >15 average seizures/day.
