# Supplementary material for: A standardized patient-centered characterization of the phenotypic spectrum of PCDH19 girls clustering epilepsy
Source: Transl Psychiatry. 2020 May 4;10:127. doi: 10.1038/s41398-020-0803-0 (PMC7198503; doi:10.1038/s41398-020-0803-0)
Supplement: Supplementary file 3 — Supplementary Materials [file 41398_2020_803_MOESM3_ESM.pdf]

Supplementary statistical output

a. Case processing based on clinical cut-off (<60) for the shift subscale on the SRS-2

Statistics

Is Shift score in clinical range

N	Valid	68
	Missing	1

Is Shift score in clinical range

		Frequency	Percent	Valid Percent	Cumulative Percent
Valid	No	10	14.5	14.7	14.7
	Yes	58	84.1	85.3	100.0
	Total	68	98.6	100.0	
Missing	System	1	1.4		
Total		69	100.0		

b. Case processing based on clinical cut-off (<60) for the total score on the SRS-2 and shift subscale severity

Case Processing Summary

	Cases					
	Valid		Missing		Total	
	N	Percent	N	Percent	N	Percent
ASD clinical range (yes/no) * Shift_Sev	68	98.6%	1	1.4%	69	100.0%

*ASD clinical range (yes/no) * Shift_Sev Crosstabulation*

Count

		Shift_Sev				
		Average (<60)	Mild (60-64)	Mod (65-70)	Severe (>70)	Total
ASD clinical range (yes/no)	Yes	10	11	9	38	68
Total		10	11	9	38	68

c. Case processing based on clinical cut-off (<60) for the inhibit subscale on the SRS-2

Statistics

Is Inhibit score in clinical range

N	Valid	68
	Missing	1

Is Inhibit score in clinical range

		Frequency	Percent	Valid Percent	Cumulative Percent
Valid	No	14	20.3	20.6	20.6
	Yes	54	78.3	79.4	100.0
	Total	68	98.6	100.0	
Missing	System	1	1.4		
Total		69	100.0		

d. Case processing based on clinical cut-off (<60) for the total score on the SRS-2 and inhibit subscale severity

Case Processing Summary

	Cases					
	Valid		Missing		Total	
	N	Percent	N	Percent	N	Percent
ASD clinical range (yes/no) * Inhibit_Sev	68	98.6%	1	1.4%	69	100.0%

*ASD clinical range (yes/no) * Inhibit_Sev Crosstabulation*

Count

		Inhibit_Sev				
		Average (<60)	Mild (60-64)	Mod (65-70)	Severe (>70)	Total
ASD clinical range (yes/no)	Yes	14	8	15	31	68
Total		14	8	15	31	68

e. Case processing based on clinical severity for the shift and inhibit subscales

Case Processing Summary

	Cases					
	Valid		Missing		Total	
	N	Percent	N	Percent	N	Percent
Shift_Sev * Inhibit_Sev	68	98.6%	1	1.4%	69	100.0%

*Shift_Sev * Inhibit_Sev Crosstabulation*

Count

		Inhibit_Sev				
		Average (<60)	Mild (60-64)	Mod (65-70)	Severe (>70)	Total
Shift_Sev	Average (<60)	2	0	3	5	10
	Mild (60-64)	4	4	2	1	11
	Moderate (65-70)	2	2	1	4	9
	Severe (>70)	6	2	9	21	38
Total		14	8	15	31	68

f. Case processing based on grouping clinical severity for the shift and inhibit subscales against the hyperactivity-inattention subscale as measured by the SDQ ("Hyp")

Case Processing Summary

	Cases					
	Valid		Missing		Total	
	N	Percent	N	Percent	N	Percent
Inhibit and shift combined severity * Hyp	56	81.2%	13	18.8%	69	100.0%

*Inhibit and shift combined severity * Hyperactivity-inattention scale from the SDQ score in clinical range (yes/no) ("Hyp") Crosstabulation*

Count

		Hyp		
		No	Yes	Total
Inhibit and shift combined severity	Shift av/mild + Inhibit mod/sev	2	8	10
	Shift mod/sev + Inhibit av/mild	4	4	8
	Shift av/mild + Inhibit av/mild	3	3	6
	Shift mod/sev + Inhibit mod/sev	6	26	32
Total		15	41	56

g. Average SDQ hyperactivity-inattention subscale score as a measure of ADHD symptom severity for individuals grouped based on clinical cut-off (<60) for SRS-2 total score as a correlate of an ASD diagnosis

Descriptive Statistics

ASD clinical (yes/no)		N	Min	Max	Mean	Std. Deviation
No	Hyperactivity-inattention scale from the SDQ	18	0	9	4.78	2.579
Yes	Hyperactivity-inattention scale from the SDQ	56	0	10	6.95	2.235

h. Average SDQ hyperactivity-inattention subscale score as a measure of ADHD symptom severity for individuals grouped based on combined shift and inhibit subscale severity

Descriptive Statistics

Inhibit and shift		N	Minimum	Maximum	Mean	Std. Deviation
Shift av/mild + Inhibit mod/sev	Hyperactivity-inattention scale from the SDQ	11	4	10	7.09	2.166
Shift mod/sev + Inhibit av/mild	Hyperactivity-inattention scale from the SDQ	9	2	10	6.00	2.550
Shift av/mild + Inhibit av/mild	Hyperactivity-inattention scale from the SDQ	19	0	10	4.37	2.692
Shift mod/sev + Inhibit mod/sev	Hyperactivity-inattention scale from the SDQ	35	4	10	7.43	1.720

i. Case processing based on ASD and ADHD total score cut-offs

Statistics

ADHD			
No	N	Valid	18
		Missing	25
Yes	N	Valid	56
		Missing	13

ADHD

		ASD (yes/no)	Frequency	Percent	Valid Percent	Cumulative Percent
No	Valid	No	10	23.3	55.6	55.6
		Yes	8	18.6	44.4	100.0
		Total	18	41.9	100.0	
	Missing	System	25	58.1		
	Total		43	100.0		
Yes	Valid	No	15	21.7	26.8	26.8
		Yes	41	59.4	73.2	100.0
		Total	56	81.2	100.0	
	Missing	System	13	18.8		
	Total		69	100.0		
