# Supplementary material for: A standardized patient-centered characterization of the phenotypic spectrum of PCDH19 girls clustering epilepsy
Source: Transl Psychiatry. 2020 May 4;10:127. doi: 10.1038/s41398-020-0803-0 (PMC7198503; doi:10.1038/s41398-020-0803-0)
Supplement: Supplementary file 4 — Supplementary Seizure Data [file 41398_2020_803_MOESM4_ESM.pdf]

Participant			Seizures				Status Epilepticus					Clustered Seizures					Isolated seizures	
ID	Group	Age	Presence	Onset (months)	Hospital admissions	ICU admission	Presence	Frequency	Duration	Convulsive	Non-convulsive	Presence	Duration (days)	Seizures/day (average)	Last 12 months	Last seizure	Presence	Frequency
1	AF	1.5	YES	8	3	YES	NO	N/A	N/A	N/A	N/A	YES	5	24	3	4/7/18	NO	N/A
2	AF	3	YES	15.5	2	YES	NO	N/A	N/A	N/A	N/A	YES	4	50	3	14/8/18	YES	Yearly
3	AF	2.5	YES	5.5	5	YES	NO	N/A	N/A	N/A	N/A	YES	1	42	14	09/07/2018	NO	N/A
4	AF	2	YES	9	3	YES	NO	N/A	N/A	N/A	N/A	UNSURE	N/A	N/A	N/A	1/10/18	NO	N/A
5	AF	2	YES	22	0	NO	NO	N/A	N/A	N/A	N/A	YES	2	4	2	25/2/18	YES	Weekly
6	MM	2.5	YES	22	1	YES	NO	N/A	N/A	N/A	N/A	YES	13	7	1	17/6/17	NO	N/A
7	AF	4	YES	6	5	YES	YES	Monthly	50-60 minutes	YES	YES	YES	3	15	6	8/6/18	NO	N/A
8	AF	2.5	YES	17	4	YES	NO	N/A	N/A	N/A	N/A	YES	10	24	4	Ongoing	YES	Twice
9	AF	4	YES	10	5	YES	NO	N/A	N/A	N/A	N/A	YES	3	17	15	24/5/18	NO	N/A
10	AF	3	YES	14	5	YES	YES	Yearly	30-50 minutes	NO	YES	YES	5	15	3	17/7/17	YES	Yearly
11	MM	2	YES	8	2	NO	NO	N/A	N/A	N/A	N/A	YES	UNSURE	UNSURE	0	1/6/16	NO	N/A
12	AF	2.5	YES	28	3	NO	NO	N/A	N/A	N/A	N/A	YES	3	8	1	10/1/18	YES	Yearly
13	AF	8	YES	6	5	YES	YES	Yearly	40-50 minutes	YES	NO	YES	4	25	12	1/1/19	YES	Infrequent
14	AF	9	YES	4	5	NO	YES	Rarely	30-40 minutes	YES	NO	YES	3	40	30	6/10/18	YES	Yearly
15	AF	7	YES	6	5	NO	YES	Yearly	30-40 minutes	YES	NO	YES	3	40	15	30/7/18	YES	Yearly
16	MM	5	YES	5	5	YES	NO	N/A	N/A	N/A	N/A	YES	2	2	5	26/8/18	YES	Monthly
17	AF	10	YES	15	5	YES	NO	N/A	N/A	N/A	N/A	YES	2	15	0	1/9/14	NO	N/A
18	MM	10	YES	11	5	NO	NO	N/A	N/A	N/A	N/A	YES	7	30	2	1/12/17	NO	N/A
19	AF	8	YES	12	4	YES	NO	N/A	N/A	N/A	N/A	YES	1	3	0	1/5/15	YES	Infrequent
20	AF	6	YES	11	4	YES	NO	N/A	N/A	N/A	N/A	YES	2	10	0	1/9/17	YES	Infrequent
21	AF	5	YES	6.5	4	YES	NO	N/A	N/A	N/A	N/A	YES	16	14	0	19/2/17	YES	Monthly
22	AF	7	YES	11	5	YES	YES	Rarely	30-40 minutes	YES	NO	YES	10	45	0	1/8/17	YES	Yearly
23	AF	10	YES	12	5	YES	YES	Bi-monthly	30-60+ minutes	NO	YES	YES	5	5	6	17/7/17	NO	N/A
24	AF	6	YES	10	3	YES	NO	N/A	N/A	N/A	N/A	YES	6	8	26	30/12/2017	YES	Infrequent
25	AF	8	YES	14	3	YES	NO	N/A	N/A	N/A	N/A	YES	2	13	0	14/12/15	YES	Monthly
26	AF	10	YES	13	5	YES	YES	Monthly-yearly	30-40 minutes	YES	NO	YES	2	100	8	21/6/18	NO	N/A
27	AF	7	YES	15	5	NO	YES	Yearly	50-60 minutes	YES	NO	YES	3	12	0	1/2/16	YES	Yearly
28	AF	9	YES	8	5	YES	NO	N/A	N/A	N/A	N/A	YES	7	10	0	1/4/17	NO	N/A
29	AF	8	YES	3	5	YES	YES	Weekly	30-60+ minutes	YES	YES	YES	4	30	1000	Ongoing	YES	Monthly
30	MM	5	YES	8	5	YES	NO	N/A	N/A	N/A	N/A	YES	3	10	24	1/3/18	NO	N/A
31	AF	6	YES	11	2	NO	NO	N/A	N/A	N/A	N/A	YES	3	5	0	6/10/15	NO	N/A
32	AF	6	YES	28	1	YES	NO	N/A	N/A	N/A	N/A	YES	14	5	0	1/1/13	NO	N/A
33	AF	8	YES	10	5	YES	YES	Daily	30-40 minutes	NO	YES	YES	3	7	5	15/6/06	NO	N/A
34	AF	13	YES	8	3	YES	NO	N/A	N/A	N/A	N/A	YES	14	20	0	1/10/08	NO	N/A
35	AF	11	YES	9	5	YES	YES	No pattern	30-50 minutes	NO	YES	YES	5	12	0	19/8/16	NO	N/A
36	AF	11	YES	8	5	NO	YES	Rarely	30-40 minutes	YES	YES	YES	4	30	0	1/9/11	YES	Monthly
37	AF	11	YES	8	5	NO	YES	Rarely	30-40 minutes	YES	YES	YES	4	30	0	1/11/11	YES	Monthly
38	AF	11	YES	6	5	YES	UNSURE	N/A	N/A	N/A	N/A	YES	7	8	6	18/2/18	YES	Monthly
39	AM(S)	16	YES	114	1	YES	NO	N/A	N/A	N/A	N/A	YES	5	2	12	1/12/18	YES	Infrequent
40	AF	15	YES	14	5	YES	NO	N/A	N/A	N/A	N/A	YES	12	28	0	1/10/08	YES	Infrequent
41	AF	12	YES	10	5	YES	YES	Once	30-40 minutes	YES	NO	YES	5	16	1	1/8/15	YES	Yearly
42	AF	15	YES	6.5	5	YES	NO	N/A	N/A	N/A	N/A	YES	2	20	11	13/01/2018	NO	N/A
43	AF	12	YES	13	5	YES	NO	N/A	N/A	N/A	N/A	YES	1	10	100	4/4/18	YES	Yearly
44	AF	16	YES	8	5	YES	YES	Daily	30-40 minutes	YES	YES	YES	4	19	12	12/8/17	YES	Unsure
45	AF	41	YES	7	2	NO	NO	N/A	N/A	N/A	N/A	YES	2	20	0	1/1/02	NO	N/A
46	AF	22	YES	8	5	NO	YES	Yearly	40-50 minutes	YES	YES	YES	5	8	0	1/1/07	YES	Yearly
47	AM(NS)	23	NO	N/A	0	N/A	N/A	N/A	N/A	N/A	N/A	N/A	N/A	N/A	N/A	N/A	N/A	N/A
48	AF	28	YES	7	5	YES	YES	Monthly	30-60+ minutes	YES	YES	YES	7	10	0	1/1/09	YES	Yearly
49	NP	35	NO	N/A	7	N/A	N/A	N/A	N/A	N/A	N/A	N/A	N/A	N/A	N/A	N/A	N/A	N/A
50	AF	30	YES	60	5	YES	NO	N/A	N/A	N/A	N/A	YES	3	10	0	1/1/97	NO	N/A
51	AF	36	YES	6	6	NO	NO	N/A	N/A	N/A	N/A	YES	1	10	0	1/1/89	YES	Yearly
52	AF	38	YES	21	6	NO	NO	N/A	N/A	N/A	N/A	YES	2	3	0	In childhood	YES	Infrequent
53	AF	35	YES	2	5	YES	YES	Once	30-40 minutes	YES	NO	NO	N/A	N/A	N/A	1/1/88	YES	Yearly
54	AF	36	YES	3	2	NO	NO	N/A	N/A	N/A	N/A	YES	2	3	8	13/3/18	YES	Yearly
55	AF	35	YES	18	2	NO	NO	N/A	N/A	N/A	N/A	YES	3	20	0	1/1/94	YES	Twice
56	NP	46	NO	N/A	7	N/A	N/A	N/A	N/A	N/A	N/A	N/A	N/A	N/A	N/A	N/A	N/A	N/A
57	AF	32	YES	24	2	YES	YES	Three times	60+ minutes	YES	YES	YES	2	8	0	1/1/02	YES	Infrequent
58	NP	33	YES	60	1	YES	NO	N/A	N/A	N/A	N/A	NO	N/A	N/A	N/A	1/1/89	YES	Once
59	AF	41	YES	10	5	NO	NO	N/A	N/A	N/A	N/A	YES	2	5	0	1/1/02	NO	N/A
60	TM	63	NO	N/A	7	N/A	N/A	N/A	N/A	N/A	N/A	N/A	N/A	N/A	N/A	N/A	N/A	N/A

61	TM	70	NO	N/A		7	N/A	N/A	N/A	N/A	N/A	N/A	N/A	N/A	N/A	N/A	N/A	N/A
62	NP	47	NO	N/A		7	N/A	N/A	N/A	N/A	N/A	N/A	N/A	N/A	N/A	N/A	N/A	N/A
63	MM(U)	41	NO	N/A		7	N/A	N/A	N/A	N/A	N/A	N/A	N/A	N/A	N/A	N/A	N/A	N/A
64	AF	31	YES	55	1	NO	NO	N/A	N/A	N/A	N/A	YES	1	7	0	1/8/98	YES	Yearly
65	TM	41	NO	N/A		7	N/A	N/A	N/A	N/A	N/A	N/A	N/A	N/A	N/A	N/A	N/A	N/A
66	AF	2	YES	11	4	NO	NO	N/A	N/A	N/A	N/A	YES	15	5	1	8/06/2018	YES	Weekly
67	MM	2.5	YES	5	5	NO	NO	N/A	N/A	N/A	N/A	YES	1	7	20	8/07/2018	NO	N/A
68	AF	3	YES	24	5	NO	NO	N/A	N/A	N/A	N/A	YES	7	10	30	1/06/2018	NO	N/A
69	AF	3	YES	8	4	NO	NO	N/A	N/A	N/A	N/A	YES	2	20	5	12/01/2018	YES	Infrequent
70	AF	2.5	YES	10	1	YES	NO	N/A	N/A	N/A	N/A	YES	6	14	6	1/02/2018	NO	N/A
71	AF	6	YES	27	1	NO	NO	N/A	N/A	N/A	N/A	YES	1	10	0	1/02/2015	NO	N/A
72	MM	7	YES	10	5	YES	NO	N/A	N/A	N/A	N/A	YES	3	15	1	1/02/2018	NO	N/A
73	AF	6	YES	14	4	YES	NO	N/A	N/A	N/A	N/A	YES	3	6	0	24/06/2015	YES	Once
74	AF	7	YES	11	3	NO	NO	N/A	N/A	N/A	N/A	YES	4	8	0	1/02/2015	YES	Infrequent
75	AF	6	YES	9	1	NO	NO	N/A	N/A	N/A	N/A	YES	3	4	0	1/08/2017	YES	Monthly
76	AF	10	YES	11	2	YES	NO	N/A	N/A	N/A	N/A	YES	2	14	0	1/08/2014	YES	Twice
77	AF	8	YES	13	5	NO	NO	N/A	N/A	N/A	N/A	YES	2	10	2	24/05/2016	YES	Once
78	AF	8	YES	8	5	YES	NO	N/A	N/A	N/A	N/A	YES	15	20	40	10/03/2018	NO	N/A
79	AF	9	YES	8	5	NO	YES	Every 3-4 months	60+ minutes	YES	YES	YES	4	20	4	1/01/2018	NO	N/A
80	AF	8	YES	6	5	NO	NO	N/A	N/A	N/A	N/A	YES	24	10	3	1/03/2018	YES	Yearly
81	AF	17	YES	5	5	NO	NO	N/A	N/A	N/A	N/A	YES	2	8	0	1/02/2016	YES	Infrequent
82	AF	17	YES	7	5	YES	YES	Once	40-50 minutes	YES	NO	YES	5	30	0	1/04/2015	YES	Yearly
83	AF	16	YES	6	5	YES	YES	Twice	30-40 minutes	YES	NO	YES	6	30	2	1/10/2017	YES	Yearly
84	AF	17	YES	15	5	YES	NO	N/A	N/A	N/A	N/A	YES	2	4	21	1/10/2018	YES	Infrequent
85	AF	13	YES	7	4	NO	NO	N/A	N/A	N/A	N/A	YES	1	25	6	21/03/2018	YES	Monthly
86	AF	15	YES	2.5	5	NO	NO	N/A	N/A	N/A	N/A	YES	14	8	0	1/01/2012	YES	Yearly
87	AF	27	YES	7	5	NO	NO	N/A	N/A	N/A	N/A	NO	N/A	N/A	N/A	30/9/18	YES	Weekly
88	AF	43	YES	9	5	NO	NO	N/A	N/A	N/A	N/A	YES	1	5	0	1/1/13	YES	Monthly
89	AF	21	YES	5	5	YES	NO	N/A	N/A	N/A	N/A	YES	1	7	2	1/10/17	NO	N/A
90	AF	21	YES	16	5	YES	NO	N/A	N/A	N/A	N/A	YES	2	10	0	24/7/03	NO	N/A
91	AF	18	YES	11	5	NO	NO	N/A	N/A	N/A	N/A	YES	3	30	0	19/02/2008	NO	N/A
92	AF	25	YES	9	5	YES	NO	N/A	N/A	N/A	N/A	NO	N/A	N/A	N/A	1/3/08	YES	Infrequent
93	NP	45	YES	3	0	NO	NO	N/A	N/A	N/A	N/A	NO	N/A	N/A	N/A	1/8/72	YES	Once
94	AF	52	YES	8	6	NO	UNSURE	N/A	N/A	N/A	N/A	YES			0	UNSURE	UNSURE	N/A
95	AF	23	YES	8	6	YES	YES	Yearly	60+ minutes	YES	YES	YES	4		0	9/5/16	YES	Once
96	TM	51	NO	N/A		7	N/A	N/A	N/A	N/A	N/A	N/A	N/A	N/A	N/A	N/A	N/A	N/A
97	NP	54	NO	N/A		7	N/A	N/A	N/A	N/A	N/A	N/A	N/A	N/A	N/A	N/A	N/A	N/A
98	AF	25	YES	30	5	NO	NO	N/A	N/A	N/A	N/A	YES	15	20	0	1/1/04	NO	N/A
99	NP	57	NO	N/A		7	N/A	N/A	N/A	N/A	N/A	N/A	N/A	N/A	N/A	N/A	N/A	N/A
100	AF	10	YES	11	5	YES	NO	N/A	N/A	N/A	N/A	YES	1	3	0	1/1/16	YES	Daily
101	AF	6	YES	16	5	NO	NO	N/A	N/A	N/A	N/A	YES	5	30	0	1/10/17	NO	N/A
102	AF	6	YES	7	5	YES	NO	N/A	N/A	N/A	N/A	YES	2	7	5	26/8/18	NO	N/A
103	AF	7	YES	3.5	5	YES	NO	N/A	N/A	N/A	N/A	YES	3	15	10	1/8/18	YES	Yearly
104	AF	5	YES	14	5	YES	YES	Twice	50-60 minutes	YES	NO	YES	2	10	5	1/3/18	NO	N/A
105	AF	7	YES	10	5	YES	NO	N/A	N/A	N/A	N/A	YES	5	10	3	16/7/18	YES	Infrequent
106	AF	14	YES	4	5	YES	NO	N/A	N/A	N/A	N/A	YES	2	6	2	1/5/18	NO	N/A
107	AF	12	YES	22	5	YES	NO	N/A	N/A	N/A	N/A	YES	7	5	0	1/1/10	NO	N/A
108	AF	32	YES	1.5	0	NO	NO	N/A	N/A	N/A	N/A	YES	4	20	0	1/2/10	NO	N/A
109	AF	23	YES	22	5	YES	NO	N/A	N/A	N/A	N/A	YES	3		0	19/12/08	YES	Twice
110	AF	23	YES	18	5	YES	NO	N/A	N/A	N/A	N/A	YES	1	Many	0	1/1/06	NO	N/A
111	AF	33	YES	18	5	NO	YES	Yearly	30-40 minutes	YES	UNSURE	YES	1	3	0	1/1/02	NO	N/A
112	MM	12	YES	96	5	NO	NO	N/A	N/A	N/A	N/A	YES	1	5	50		YES	Yearly
