# Supplementary material for: A standardized patient-centered characterization of the phenotypic spectrum of PCDH19 girls clustering epilepsy
Source: Transl Psychiatry. 2020 May 4;10:127. doi: 10.1038/s41398-020-0803-0 (PMC7198503; doi:10.1038/s41398-020-0803-0)
Supplement: Supplementary file 5 — Supplementary Tables [file 41398_2020_803_MOESM5_ESM.pdf]

Supplementary Tables

Table 1. Reliability coefficients for SDQ Parent-Report scores

		Cronbach α
SDQ scale	Total difficulties	.71
	Emotional symptoms	.77
	Conduct problems	.63
	Hyperactivity-inattention	.67
	Peer problems	.66
	Prosocial behavior	.83
	Impact	.86

SDQ Strengths and Difficulties Questionnaire

Table 2. *In silico* assessment of all novel *PCDH19* variants in our cohort

	#	Country	Position	NM_001184880.1	Mode of inheritance	CADD (P≥25)	gnomAD frequency	GPP (P ≥0.1)	Mut Pred rank (P≥0.68)	MutationAssessor rank (P≥0.59)	PROVEAN (P≤-2.5)	Polyphen2	SIFT	Prediction
Missense	7	Denmark	99661786	c.1810A>C; p.Thr604Pro	*De novo*	24.2	Absent	0.1560795	0.722	0.895	-3.68	D	Del	Likely Pathogenic
	8	Australia	99663098-99663100	c.496_498AAA; p.Tyr166Lys	*De novo*	NC	Absent	NC	0.896	NC	-8.199	D	NC	Likely Pathogenic
	16	Moldova	99663003	c.593G>C; p.Arg198Pro (mosaic)	*De novo*	31	Absent	0.4938962	0.927	0.987	-6.44	D	Del	Likely Pathogenic
	23	USA	99662127	c.1469A>C; p.Tyr490Ser	*De novo*	25.6	Absent	0.9081805	0.919	0.991	-8.43	D	Del	Likely Pathogenic
	26,56	Denmark	99662797	c.799G>A; p.Glu267Lys	Maternal, Paternal	33	Absent	0.1402426	0.887	0.771	-3.59	D	Del	Likely Pathogenic
	27	Denmark	99662127	c.1469A>G; p.Tyr490Cys	Paternal	24.5	Absent	0.8659184	0.908	0.978	-8.43	D	Del	Likely Pathogenic
	30	UK	99662908	c.688G>C; p.Asp230His (mosaic)	*De novo*	27.5	Absent	0.9995749	0.888	0.999	-6.19	D	Del	Likely Pathogenic
	40	New Zealand	99661677	c.1919T>G; p.Leu640Arg	*De novo*	25.1	Absent	0.0750358	0.87	0.972	-5.26	D	Del	Likely Pathogenic
	44	USA	99662994	c.602A>C; p.Gln201Pro	*De novo*	24.4	Absent	0.1126678	0.686	0.897	-4.9	D	Del	Likely Pathogenic
	67	Italy	99662576	c.1020T>A; p.Asn340Lys (mosaic)	*De novo*	23.9	Absent	0.5263762	0.915	0.998	-5.51	D	Del	Likely Pathogenic
	69	Italy	99661654	c.1942G>C; p.Gly648Arg	*De novo*	28.6	Absent	0.9850428	0.866	0.913	-7.06	D	Del	Likely Pathogenic
	73	Italy	99662925	c.671T>A; p.Leu224His	*De novo*	24.5	Absent	0.9962677	0.81	0.94	-5.3	D	Del	Likely Pathogenic
	78	Italy	99551837	c.2885G>A; p.Arg962Gln	Unknown	32	0.0000056 (1 x male)	0.0001695	0.657	0.758	-1.92	D	Del	Likely Pathogenic
	85	Italy	99662817	c.779T>G; p.Leu260Arg	*De novo*	25.8	Absent	0.9746687	0.959	0.959	-5.53	D	Del	Likely Pathogenic
	89,97, 98,99	Italy	99661623	c.1973T>G; p.Val658Gly	Maternal (3) Unknown	23.4	Absent	0.8591691	0.587	0.819	-4.08	P	Del	Likely Pathogenic
	90,96	Italy	99662133	c.1463T>A; p.Val488Asp	Paternal, Unknown	26	Absent	0.3091389	0.912	0.987	-5.88	D	Del	Likely Pathogenic
	91	Italy	99663460	c.136G>C; p.Ala46Pro	Paternal	25.3	Absent	5.81E-05	0.884	0.355	-1.5	D	Tol	Likely Pathogenic
	111	France	99662628	c.968C>T; p.Pro323Leu	Paternal	26.1	Absent	0.5317213	0.803	0.132	-8.44	D	Del	Likely Pathogenic
	Ex	USA	99658583	c.2227T>A; p.Ser743Thr	Maternal (2), Unknown	6.798	0.0000115	8.40E-10	0.157	0.065	0.14	B	Tol	Likely Benign
Nonsense	11	Argentina	99661876	c.1720G>T; p.Glu574* (mosaic)	*De novo*	37	Absent	NC	NC	NC	NC	NC	NC	Likely Pathogenic
	59	Netherlands	99657726	c.2412C>A; p.Cys804*	Unknown	38	Absent	NC	NC	NC	NC	NC	NC	Likely Pathogenic
	70	Italy	99662844	c.752C>A; p.Ser251*	*De novo*	41	Absent	NC	NC	NC	NC	NC	NC	Likely Pathogenic
	107	France	99663544	c.52C>T; p.Gln18*	*De novo*	36	Absent	NC	NC	NC	NC	NC	NC	Likely Pathogenic
	112	USA	99662048	c.1548C>A; p.Tyr516*	Unknown	32	Absent	NC	NC	NC	NC	NC	NC	Likely pathogenic
Frameshift	3	Russia	99662500	c.1095_1096insG; p.Tyr366Valfs*10	*De novo*	NC	Absent	NC	NC	NC	NC	NC	NC	Possibly Damaging
	4,52	Russia	99662982	c.614del; p.Ser205Thrfs*7	Maternal, *De novo*	NC	Absent	NC	NC	NC	NC	NC	NC	Possibly Damaging
	35	USA	99663061-99663088	c.518_525del; p.Leu173Profs*50	*De novo*	NC	Absent	NC	NC	NC	NC	NC	NC	Possibly Damaging
	66	Italy	99662139	c.1457del; p.Gly486Alafs*83	*De novo*	NC	Absent	NC	NC	NC	NC	NC	NC	Possibly Damaging
	71	Italy	99661880-99661886	c.1710_1716del; p.Asn570Lysfs*12	*De novo*	NC	Absent	NC	NC	NC	NC	NC	NC	Possibly Damaging
	87	Italy	99662437	c.1159delC; p.Arg387Valfs*135	Unknown	NC	Absent	NC	NC	NC	NC	NC	NC	Possibly Damaging
	100	France	99662625	c.971del; p.Asn324Ilefs*44	Maternal	NC	Absent	NC	NC	NC	NC	NC	NC	Possibly Damaging
	101	France	99661637-99661638	c.1958_1959del; p.Ser653Cysfs*64	*De novo*	NC	Absent	NC	NC	NC	NC	NC	NC	Possibly Damaging
	104	Canada	99657637	c.2501dup; p.Asn834Lysfs*13	Unknown	NC	Absent	NC	NC	NC	NC	NC	NC	Possibly Damaging
	110	France	99662851	c.745del; p.Glu249Lysfs*56	*De novo*	NC	Absent	NC	NC	NC	NC	NC	NC	Possibly Damaging
Dup	5,10, 64	USA	99663146-99663151	c.445_450dup; p.Pro149_Leu150dup	Maternal (2), Unknown	NC	Absent	NC	NC	NC	-9.85	NC	NC	Possibly Damaging
Splice	22	UK	99551874	c.2849-1G>C; p.?	Unknown	33	Absent	NC	NC	NC	NC	NC	NC	Likely Pathogenic

D probably damaging, *P* possibly damaging, *B* benign, *Del* deleterious, *Tol* tolerated, *N* neutral, *L* low, *M* medium, *H* high, *Dup* duplication, *NC* not covered, NB: red text highlights benign scores, *Ex* refers to the excluded variant

Table 3. *In silico* assessment of all non-*PCDH19* variants in our cohort

	Participant	PCDH19	Secondary findings	Position	Mode of inheritance	CADD (P≥25)	gnomAD frequency	Mut Pred rank (P≥0.68)	MutationAssessor rank (P≥0.59)	PROVEAN (P≤-2.5)	Polyphen2	SIFT	Prediction
Non-PCDH19	1	c.1091dup; p.Tyr366Leufs*10	STRADA: c.1144-1G>A; p.?	63703741	Unknown	NC	Absent	NC	NC	NC	NC	NC	Possibly Damaging
			TPK1: c.337G>A:p.Glu113Lys	144320276	Unknown	17.53	Absent	0.2	0.094	-0.04	B	Tol	Likely Benign
	2	c.1335C>G; p.Asp445Glu	*KANSL1*: c.53T>C; p.Ile8Thr	44249457	Unknown	24.8	Absent	0.362	0.225	-0.117647059	D	Del	Possibly Damaging
	5#	c.445_450dup; p.Pro149_Leu150dup	*CHRNA4*: c.1183G>A:p.Val395Ile	61981052	Maternal	15.1	0.0002254	0.15	0.646	-0.8	D	Tol	Likely Benign
	6	c.2656C>T; p.Arg886* (mosaic)	WWOX: c.1057C>A:p.Gln353Lys	79245505	*De novo*	23.3	0.00001206	0.71	NC	-1.72	P	Tol	Likely Benign
			ZEB2: c.225C>T:p.Ser75=	145187442	*De novo*	NC	0.00003186	NC	NC	NC	NC	NC	Likely Benign
	10#	c.445_450dup; p.Pro149_Leu150dup	*CHRNA4*: c.1183G>A:p.Val395Ile	61981052	Maternal	15.1	0.0002254	0.15	0.646	-0.8	D	Tol	Likely Benign
	30	c.688G>C; p.Asp230His (mosaic)	*SCN9A*: c.2215A>G:p.Ile739Val	167136962	*De novo*	18.02	0.00247	0.654	0.8	-0.89	P	Del	Likely Benign
	64#	c.445_450dup; p.Pro149_Leu150dup	*CHRNA4*: c.1183G>A:p.Val395Ile	61981052	Unknown	15.1	0.0002254	0.15	0.646	-0.8	D	Tol	Likely Benign

CADD Combined Annotation Dependent Depletion, *PROVEAN* Protein Variation Effect Analyzer, *Polyphen2* Polymorphism Phenotyping v2, *SIFT* Sorting Intolerance from Tolerance, *D* probably damaging, *P* possibly damaging, *B* benign, *Del* deleterious, *Tol* tolerated, *N* neutral, *NC* not covered, NB: red text highlights benign scores and # represent a mother and her two daughters

Table 4. Development frequencies (and percentages)

a. Early development based on parent/caregiver-report (*n* = 83)

	Heterozygous females (%) *n* = 73	Mosaic males (%) *n* = 8	Hemizygous Males (%) *n* = 2
**Developmental delay**	43/73 (59)	6/8 (75)	1/2 (50)
-Prior to seizure onset	13/43 (30)	2/6 (33)	N/A
**Regression**	31/73 (42)	4/8 (50)	2/2 (100)
- ≥5 episodes of regression	17/31 (55)	3/4 (75)	1/2 (50)
-Following seizure cluster	27/31 (87)	2/4 (50)	1/2 (50)
-Following status epilepticus	5/31 (16)	0/4 (0)	0/2 (0)

b. Intellect based on self- or patient/caregiver-report (*n* = 111)

	Heterozygous females		Mosaic males		Hemizygous males	
	Affected (*n* = 89)	Non-penetrant (*n* = 7)	Affected (*n* = 8)	Non-penetrant (*n* = 1)	Affected (*n* = 2)	Transmitting (*n* = 4)
Normal intelligence	45	7	4	1	1	4
Borderline intelligence	3	0	1	0	0	0
Mild ID	18	0	1	0	1	0
Moderate ID	9	0	0	0	0	0
Severe ID	12	0	2	0	0	0
Profound ID	2	0	0	0	0	0

ID intellectual disability

Table 5. Frequencies (and percentages) of Strengths and Difficulties Questionnaire (SDQ) domain and impact scores for affected individuals

a. Heterozygous females (*n* = 65)

	SDQ Domains				
SDQ score classification	Emotional Problems	Conduct Problems	Hyperactive-Inattention	Peer Problems	Prosocial Behavior
Average	35 (54)	25 (38.5)	22 (34)	19 (29.5)	17 (26)
Mild	12 (18)	13 (20)	15 (23)	6 (9)	9 (14)
Moderate	7 (11)	16 (24.5)	11 (17)	6 (9)	7 (11)
Severe	11 (17)	11 (17)	17 (26)	34 (52.5)	32 (49)

b. Mosaic males (*n* = 8)

	SDQ Domains				
SDQ score classification	Emotional Problems	Conduct Problems	Hyperactive-Inattention	Peer Problems	Prosocial Behavior
Average	6 (75)	4 (50)	2 (25)	2 (25)	0 (0)
Mild	0 (0)	2 (25)	3 (37.5)	0 (0)	2 (25)
Moderate	1 (12.5)	1 (12.5)	2 (25)	2 (25)	0 (0)
Severe	1 (12.5)	1 (12.5)	1 (12.5)	4 (50)	6 (75)

c. Impact scores (*n* = 73)

SDQ score classification	Heterozygous females (*n* = 65)	Mosaic Males (*n* = 8)
Average	9 (14)	1 (12.5)
Mild	6 (9)	1 (12.5)
Moderate	3 (5)	0 (0)
Severe	47 (72)	6 (75)

Table 6. Descriptive statistics for all neuropsychiatric measures

Group	Measure	N	Min	Max	Mean	SD
Heterozygous females	SRS-2	82	35	107	69.5*	18.6
	SCQ	8	8	32	18.5*	8.12
	SDQ	65	3	31	17.6*	6.68
	BRIEF	89	34	98	67.8*	15.8
	DOCS	17	0	33	11.4	10.7
Mosaic males	SRS-2	8	46	86	68.8*	15.1
	SDQ	8	8	29	16.5*	6.28
	BRIEF	8	51	80	63.9*	11.7
Non-penetrant females	SRS-2	7	41	70	47.7	10.1
	BRIEF	7	39	75	47.6	12.5
	DOCS	7	0	46	11.1	16.3
Transmitting males	SRS-2	4	41	51	46.3	4.99
	BRIEF	4	38	53	45.3	6.19
	DOCS	4	0	15	5.25	6.70

***Represents group averages above the clinical threshold**

Table 7. Average scores on measures of executive dysfunction and ASD based on seizure onset and activity

Group	*N*	BRIEF GEC *t* score	SRS-2/SCQ combined *z* score
Late / Mild	22	61.0	-0.35
Late / Severe	6	63.7	0.01
Early / Mild	33	71.8	0.17
Early / Severe	24	75.3	0.85

Table 8. Genotype-phenotype associations

**Dependent variable**	**Independent variable**	**N**	**Mean**	**Std. Deviation**	**Significance**
BRIEF *t* score	Non-truncating	59	61.8	16.1	$p = .067$
	Truncating	38	68.1	17.0	
ASD *z* score (SRS-2 & SCQ)	Non-truncating	59	-0.12	0.98	$p = .738$
	Truncating	38	-0.05	1.04	
SCQ emotional problems	Non-truncating	29	3.62	2.77	$p = .128$
	Truncating	32	2.63	2.27	
SCQ conduct problems	Non-truncating	29	3.41	2.01	$p = .941$
	Truncating	32	3.38	2.03	
SCQ hyperactivity-inattention	Non-truncating	29	6.52	2.23	$p = .491$
	Truncating	32	6.06	2.83	
SCQ peer problems	Non-truncating	29	4.48	2.17	$p = .190$
	Truncating	32	3.66	2.70	
SCQ prosocial behavior	Non-truncating	29	5.21	2.87	$p = .545$
	Truncating	32	4.75	2.98	
Age at seizure onset	Non-truncating	48	10.3	6.35	$p = .506$
	Truncating	34	11.2	6.20	
