# Supplementary material for: A standardized patient-centered characterization of the phenotypic spectrum of PCDH19 girls clustering epilepsy
Source: Transl Psychiatry. 2020 May 4;10:127. doi: 10.1038/s41398-020-0803-0 (PMC7198503; doi:10.1038/s41398-020-0803-0)
Supplement: Supplementary file 6 — Supplementary Variant Data [file 41398_2020_803_MOESM6_ESM.pdf]

Participant	Group	Variant	Inheritance	Novel	Relatives
1	Heterozygous female	c.1091dup; p.Tyr366Leufs*10	Unknown		
2	Heterozygous female	c.1335C>G; p.Asp445Glu	Unknown		
3	Heterozygous female	c.1095_1096insG; p.Tyr366Valfs*10	De novo	Yes	
4	Heterozygous female	c.614del; p.Ser205Thrfs*7	Maternal	Yes	Yes (52)
5	Heterozygous female	c.445_450dup; p.Pro149_Leu150dup	Maternal	Yes	Yes (10,64)
6	Mosaic male	c.2656C>T; p.Arg886*	De novo		
7	Heterozygous female	c.1810A>C; p.Thr604Pro	De novo	Yes	
8	Heterozygous female	c.496_498AAA; p.Tyr166Lys	De novo	Yes	
9	Heterozygous female	c.370G>A; p.Asp124Asn	Maternal		Yes (42,45,53,61,62)
10	Heterozygous female	c.445_450dup; p.Pro149_Leu150dup	Maternal	Yes	Yes (5,64)
11	Mosaic male	c.1720G>T; p.Glu574*	De novo	Yes	
12	Heterozygous female	c.1019A>G; p.Asn340Ser	Maternal		Yes (54)
13	Heterozygous female	c.1335C>A; p.Asp445Glu	Maternal		Yes (49)
14	Heterozygous female	c.1091dup; p.Tyr366Leufs*10	Paternal		
15	Heterozygous female	c.1873A>G; p.Arg625Gly	Maternal		Yes (51)
16	Mosaic male	c.593G>C; p.Arg198Pro	De novo	Yes	
17	Heterozygous female	WGD	De novo		
18	Mosaic male	c.2147+2T>C; p.?	De novo		
19	Heterozygous female	c.2113C>T; p.Arg705*	De novo		
20	Heterozygous female	c.2146dup; p.Ser716Lysfs*2	Paternal		
21	Heterozygous female	c.1114C>T; p.Arg372Trp	De novo		
22	Heterozygous female	c.2849-1G>C; p.?	Unknown	Yes	
23	Heterozygous female	c.1469A>C; p.Tyr490Ser	De novo	Yes	
24	Heterozygous female	c.498C>G; p.Tyr166*	Maternal		Yes (58)
25	Heterozygous female	c.2341dup; p.Ile781Asnfs*3	De novo		
26	Heterozygous female	c.799G>A; p.Glu267Lys	Maternal	Yes	Yes (56)
27	Heterozygous female	c.1469A>G; p.Tyr490Cys	Paternal	Yes	
28	Heterozygous female	c.1240G>A; p.Glu414Lys	Paternal		Yes (63)
29	Heterozygous female	c.1091dup; p.Tyr366Leufs*10	De novo		
30	Mosaic male	c.688G>C; p.Asp230His	De novo	Yes	
31	Heterozygous female	c.1683_1696del; p.Val562Thrfs*4	Paternal		Yes (65)
32	Heterozygous female	WGD	De novo		
33	Heterozygous female	WGD	De novo		
34	Heterozygous female	WGD	Unknown		
35	Heterozygous female	c.518_525del; p.Leu173Profs*50	De novo	Yes	
36	Heterozygous female	c.497dup; p.Tyr166*	De novo		Yes (37)
37	Heterozygous female	c.497dup; p.Tyr166*	De novo		Yes (36)
38	Heterozygous female	c.593G>T; p.Arg198Leu	De novo		
39	Hemizygous male (with epilepsy)	c.1672G>C; p.Asp558His	Unknown		
40	Heterozygous female	c.1919T>G; p.Leu640Arg	De novo	Yes	
41	Heterozygous female	c.1091dup; p.Tyr366Leufs*10	Paternal		
42	Heterozygous female	c.370G>A; p.Asp124Asn	Maternal		Yes (9,45,53,61,62)
43	Heterozygous female	c.361G>C; p.Asp121His	De novo		
44	Heterozygous female	c.602A>C; p.Gln201Pro	De novo	Yes	
45	Heterozygous female	c.370G>A; p.Asp124Asn	Paternal		Yes (9,42,53,61,62)
46	Heterozygous female	c.74T>C; p.Leu25Pro	Maternal		Yes (48)
47	Hemizygous male (no epilepsy)	c.2341dup; p.Ile781Asnfs*3	Maternal		
48	Heterozygous female	c.74T>C; p.Leu25Pro	Maternal		Yes (46)
49	Non-penetrant female	c.1335C>A; p.Asp445Glu	Unknown		Yes (13)
50	Heterozygous female	c.437C>G; p.Thr146Arg	Paternal		Yes (108,109)
51	Heterozygous female	c.1873A>G; p.Arg625Gly	Unknown		Yes (15)
52	Heterozygous female	c.614del; p.Ser205Thrfs*7	De novo	Yes	Yes (4)
53	Heterozygous female	c.370G>A; p.Asp124Asn	Paternal		Yes (9,42,45,61,62)
54	Heterozygous female	c.1019A>G; p.Asn340Ser	Unknown		Yes (12)
55	Heterozygous female	c.1671C>G; p.Asn557Lys	Paternal		Yes (57,60)
56	Non-penetrant female	c.799G>A; p.Glu267Lys	Paternal	Yes	Yes (26)
57	Heterozygous female	c.1671C>G; p.Asn557Lys	Paternal		Yes (55,60)
58	Non-penetrant female	c.498C>G; p.Tyr166*	Unknown		Yes (24)
59	Heterozygous female	c.2412C>A; p.Cys804*	Unknown	Yes	
60	Transmitting male	c.1671C>G; p.Asn557Lys	Unknown		Yes (55,57)
61	Transmitting male	c.370G>A; p.Asp124Asn	Maternal		Yes (9,42,45,53,62)
62	Non-penetrant female	c.370G>A; p.Asp124Asn	Paternal		Yes (9,42,45,53,61)
63	Non-penetrant mosaic male	c.1240G>A; p.Glu414Lys	De novo		Yes (28)
64	Heterozygous female	c.445_450dup; p.Pro149_Leu150dup	Unknown	Yes	Yes (5,10)
65	Transmitting male	c.1683_1696del; p.Val562Thrfs*4	Unknown		Yes (31)
66	Heterozygous female	c.1457del; p.Gly486Alafs*83	De novo	Yes	
67	Mosaic male	c.1020T>A; p.Asn340Lys	De novo	Yes	
68	Heterozygous female	c.2338A>T; p.Lys780*	De novo		
69	Heterozygous female	c.1942G>C; p.Gly648Arg	De novo	Yes	
70	Heterozygous female	c.752C>A; p.Ser251*	De novo	Yes	
71	Heterozygous female	c.1710_1716del; p.Asn570Lysfs*12	De novo	Yes	
72	Mosaic male	c.1352C>T; p.Pro451Leu	De novo		
73	Heterozygous female	c.671T>A; p.Leu224His	De novo	Yes	
74	Heterozygous female	c.1098C>G; p.Tyr366*	De novo		
75	Heterozygous female	c.2873G>A; p.Arg958Gln	Maternal		Yes (93)
76	Heterozygous female	c2617-1G>A; p.?	De novo		
77	Heterozygous female	c.1178C>T; p.Pro393Leu	De novo		
78	Heterozygous female	c.2885G>A; p.Arg962Gln	Unknown	Yes	
79	Heterozygous female	WGD	De novo		
80	Heterozygous female	c.1091dup; p.Tyr366Leufs*10	De novo		
81	Heterozygous female	c.1019A>G; p.Asn340Ser	De novo		
82	Heterozygous female	c.1129G>C; p.Asp377His	De novo		
83	Heterozygous female	c.2675-6A>G; p.?	De novo		
84	Heterozygous female	WGD	De novo		
85	Heterozygous female	c.779T>G; p.Leu260Arg	De novo	Yes	
86	Heterozygous female	c.958dup; p.Asp320Glyfs*22	De novo		
87	Heterozygous female	c.1159delC; p.Arg387Valfs*135	Unknown	Yes	
88	Heterozygous female	c.706C>T; p.Pro236Ser	De novo		
89	Heterozygous female	c.1973T>G; p.Val658Gly	Maternal	Yes	Yes (97,98,99)
90	Heterozygous female	c.1463T>A; p.Val488Asp	Paternal	Yes	Yes (96)
91	Heterozygous female	c.136G>C; p.Ala46Pro	Paternal	Yes	
92	Heterozygous female	c.1298T>C; p.Leu433Pro	De novo		
93	Non-penetrant female	c.2873G>A; p.Arg958Gln	Unknown		Yes (75)
94	Heterozygous female	c.1019A>G; p.Asn340Ser	De novo		Yes (95)
95	Heterozygous female	c.1019A>G; p.Asn340Ser	Maternal		Yes (94)
96	Transmitting male	c.1463T>A; p.Val488Asp	Unknown	Yes	Yes (90)
97	Non-penetrant female	c.1973T>G; p.Val658Gly	Maternal	Yes	Yes (89,98,99)
98	Heterozygous female	c.1973T>G; p.Val658Gly	Maternal	Yes	Yes (89,97,99)
99	Non-penetrant female	c.1973T>G; p.Val658Gly	Unknown	Yes	Yes (89,97,98)
100	Heterozygous female	c.971del; p.Asn324Ilefs*44	Maternal	Yes	
101	Heterozygous female	c.1958_1959del; p.Ser653Cysfs*64	De novo	Yes	
102	Heterozygous female	c.1091del; p.Pro364Argfs*4	De novo		
103	Heterozygous female	c.2656C>T; p.Arg886*	De novo		
104	Heterozygous female	c.2501dup; p.Asn834Lysfs*13	Unknown	Yes	
105	Heterozygous female	c.1091dup; p.Tyr366Leufs*10	De novo		
106	Heterozygous female	c.2019del; p.Ser674Leufs*2	De novo		
107	Heterozygous female	c.52C>T; p.Gln18*	De novo	Yes	
108	Heterozygous female	c.437C>G; p.Thr146Arg	Paternal		Yes (50,109)
109	Heterozygous female	c.437C>G; p.Thr146Arg	Paternal		Yes (50,108)
110	Heterozygous female	c.745del; p.Glu249Lysfs*56	De novo	Yes	
111	Heterozygous female	c.968C>T; p.Pro323Leu	Paternal	Yes	
112	Mosaic male	c.1548C>A; p.Tyr516*	Unknown	Yes	
